# Effects of nicotinic acetylcholine receptor-activating alkaloids on anxiety-like behavior in zebrafish

**DOI:** 10.1007/s11418-021-01544-8

**Published:** 2021-07-15

**Authors:** Ainhoa Alzualde, Oihane Jaka, Diogo A. R. S. Latino, Omar Alijevic, Iñaki Iturria, Jorge Hurtado de Mendoza, Pavel Pospisil, Stefan Frentzel, Manuel C. Peitsch, Julia Hoeng, Kyoko Koshibu

**Affiliations:** 1 Biobide, Gipuzkoa Scientific & Technological Park, 20009 San Sebastian, Spain; 2 PMI R&D, Philip Morris Products S.A, Quai Jeanrenaud 5, 2000 Neuchâtel, Switzerland; 3 Gestión de Recursos E Innovación S.L. (Grilab) C/ Américo, Castro 94, 1ºC, 28050 Madrid, Spain

**Keywords:** Alkaloids, Nicotine, Nicotinic acetylcholine receptors, Zebrafish, Anxiety

## Abstract

Alkaloids are a structurally complex group of natural products that have a diverse range of biological activities and significant therapeutic applications. In this study, we examined the acute, anxiolytic-like effects of nicotinic acetylcholine receptor (nAChR)-activating alkaloids with reported neuropharmacological effects but whose effects on anxiety are less well understood. Because α4β2 nAChRs can regulate anxiety, we first demonstrated the functional activities of alkaloids on these receptors in vitro. Their effects on anxiety-like behavior in zebrafish were then examined using the zebrafish novel tank test (NTT). The NTT is a relatively high-throughput behavioral paradigm that takes advantage of the natural tendency of fish to dive down when stressed or anxious. We report for the first time that cotinine, anatabine, and methylanatabine may suppress this anxiety-driven zebrafish behavior after a single 20-min treatment. Effective concentrations of these alkaloids were well above the concentrations naturally found in plants and the concentrations needed to induce anxiolytic-like effect by nicotine. These alkaloids showed good receptor interactions at the α4β2 nAChR agonist site as demonstrated by in vitro binding and in silico docking model, although somewhat weaker than that for nicotine. Minimal or no significant effect of other compounds may have been due to low bioavailability of these compounds in the brain, which is supported by the in silico prediction of blood–brain barrier permeability. Taken together, our findings indicate that nicotine, although not risk-free, is the most potent anxiolytic-like alkaloid tested in this study, and other natural alkaloids may regulate anxiety as well.

**Supplementary Information:**

The online version contains supplementary material available at 10.1007/s11418-021-01544-8.

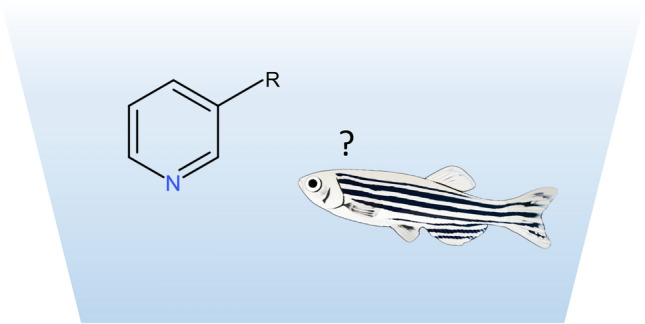

## Introduction

Plants are a rich source of nutrients and chemical ingredients that help maintain physical and mental health [45]. Alkaloids are one such class of nitrogen containing natural organic chemicals that are widely distributed throughout the plant kingdom with many reported health benefits [45, 46]. Plants, bacteria, fungi, and even animals have been found to contain alkaloids in around 15% of cases [69]. Some plant families contain more alkaloid-containing taxa than others, such as the poppy family Papaveraceae, dogbane family Apocynaceae, daisy family Asteraceae, lily family Liliaceae, buttercup family Ranunculaceae, and nightshade family Solanaceae [69]. Several food plants and products also contain alkaloids, such as comfrey, honey, coffee, rye, wheat, and barley [69].

Among alkaloids, those that activate nicotinic acetylcholine receptors (nAChRs) are of great interest due to the critical role nAChRs play in regulating neuropharmacology of mood and anxiety [48, 63]. Nicotinic AChRs are composed of α (α1–α10), β (β1–β4), and other (δ, γ, ε) subunits, which combine to form ligand-gated pentameric cation channels. In the brain and spinal cord, the homomeric α7 and heteromeric α4β2 nAChRs are the best characterized and most abundant subtypes [21]. Other nAChRs in the brain can contain α3, α5, α6, β3, and β4 subunits in various combinations [20, 21]. The behavioral complexity caused by nicotinic compounds is thought to be due to the large number of nAChRs with different time courses of activation and sensitization that exists in various cell types involved in modulating a broad range of neurotransmitter systems [47]. Preclinical animal research and clinical trials both indicate that drugs that influence nAChR activity can affect mood and anxiety-related behaviors [40, 47, 48, 72]. In fact, several α4β2 nAChR agonists (e.g., TC-2216, sazetidine) induce anti-depressant- and anxiolytic-like effects in rodents [52, 64, 72].

In this study, we investigated the behavioral and pharmacological properties of seven nAChR-activating alkaloids, previously identified to be present in Solanaceous plants [4], to understand their effects on anxiety. Six alkaloids—cotinine, anatabine, methylanatabine, anabasine, nornicotine, and metanicotine—were selected due to their chemical similarities with nicotine, a well-established natural alkaloid that can fully activate α4β2 nAChR and regulate memory and anxiety in rodents and humans [2, 8, 31, 61, 63, 70]. We selected cotinine and nornicotine, because they are also major and minor metabolites of nicotine, respectively [19]. Cotinine, in particular, has been reported to regulate specific types of memory [13, 62]. There are also sparse publications reporting the effects of anatabine, metanicotine, and anabasine on memory functions [34, 35]. However, their effects on anxiety are yet unclear. Reference compounds, acetylcholine and α4β2 nAChR agonist AZD1446, were tested to understand the effects of endogenous or synthetic nAChR agonists on anxiety-like behavior in zebrafish, which has not been assessed in previous studies. Buspirone, a clinical anxiolytic drug, and nicotine served as positive controls to validate the behavioral paradigm, the zebrafish novel tank test (NTT).

The zebrafish NTT was selected as a relatively high-throughput behavioral test to determine the anxiolytic-like effects of the compounds. During the past few decades, zebrafish have emerged as a model vertebrate organism for analyzing complex molecular and cellular interactions in vivo with some reported translational relevance to humans [16, 32, 43, 49, 58, 60]. More specifically, zebrafish have long been recognized as a valuable animal model for neurobehavioral studies, and mounting evidence indicates that they are capable of modeling a number of anxiety-related conditions [26, 30, 58]. The NTT takes advantage of the innate behavior of zebrafish to dive and dwell at the bottom of a body of water to avoid danger or stress. This behavioral paradigm has been previously validated by many labs to test the anxiolytic-like effects of compounds, such as nicotine, fluoxetine, diazepam, buspirone, chlordiazepoxide, and tranylcypromine [5, 6, 10, 14, 18, 22, 25, 26, 29, 30, 32, 36, 37, 39, 44, 50, 53, 57–59, 66]. In fact, regulation of this anxiety-like behavior by nicotine and other nicotinic ligands has been reported in several publications, supporting the pharmacological relevance of nAChR in zebrafish anxiety [5, 28, 32, 33, 53, 67].

We report here that nicotine, cotinine, anatabine, and methylanatabine have the potential to reduce anxiety-like behavior in zebrafish, while other nAChR-targeting alkaloids, such as anabasine, nornicotine, and metanicotine, have no effect. To support our behavioral findings, nicotine, cotinine, anatabine and methylanatabine were docked computationally on nAChR α4β2 crystal structure. Results show that the binding affinity of the docked alkaloids is in agreement with the in vitro nAChR α4β2 functional and binding assays. The alkaloids’ brain bioavailability varied, which was primarily confirmed by differences in blood–brain barrier permeability predicted by in silico models. These additional findings provided further explanations why the alkaloids examined in this study may have induced different levels of anxiolytic-like effects.

## Materials and methods

### Animals

Wild-type zebrafish (*Danio rerio*; strain AB) were bred and housed at Biobide (San Sebastián, Spain) in accordance with standard procedures (Zebrafish Information Network) as described previously [3, 51]. In brief, the fish were maintained in a 300-L aquarium with a maximum of 1000 fish per tank. System water was maintained at 28.5 ºC, pH 7–7.8, conductivity at 500–800 μS, and > 85% oxygen saturation and continuously filtered. The system water condition was monitored daily and regulated, if required. The fish were kept under a 14-/10-h light/dark cycle (light on at 7:30 am). Adults were fed ground dry pellets (Gemma Micro 300; Skretting Zebrafish, Westbrook, ME, USA) and live food (Artemia; Catvis B.V.,'s-Hertogenbosch, Netherlands) once a day. All behavioral experiments were performed on male and female adult zebrafish (approximately 36–52 weeks post-fertilization) in accordance with European standards of animal welfare on animal use for scientific purposes (2010/63/EU), complied with national regulations for the care of experimental animals, and were approved as described in national regulations (RD 53/2013) by local and regional committees: PRO-AE-SS-121 and PRO-AE-SS-134.

### Chemicals

Acetylcholine bromide (CAS No. 66-23-9), (–)-nicotine free base (CAS No. 54-11-5), (–)-cotinine free base (CAS No. 486-56-6), and ( ±)-nornicotine (CAS No. 5746-86-1) were purchased from Sigma-Aldrich (St. Louis, MO, USA). ( +)-Anabasine hydrochloride (CAS No. 53912-89-3), buspirone hydrochloride (CAS No. 33386-08-2), and metanicotine oxalate (CAS No. 220662-95-3) were purchased from Tocris Bioscience (Bio-Techne^®^, Minneapolis, MN, USA). ( ±)-Anatabine free base, AZD1446, and (S)-N-methylanatabine dihydrochloride were custom synthesized by WuXi AppTec (all: purity ≥ 95%; Shanghai, China).

### Zebrafish NTT

The NTT conditions closely matched the most commonly used conditions described by [32]. In brief, adult male and female wild-type zebrafish were treated with the compounds for 20 min in a final volume of 50 mL in a 250-mL treatment beaker, one fish at a time. The fish were briefly rinsed in fresh system water, and then immediately transferred to a trapezoidal tank (14.6-cm height × 5.5-cm width × 27.9-cm top length and × 23.6-cm bottom length) filled with 1.5 L system water. The behavior of the fish was monitored for the next 5 min using the Noldus EthoVision XT system (Wageningen, Netherlands), with the camera placed approximately 1 m from the test tank. The tanks were uniformly illuminated from above at approximately 200 lx, reported to be the optimal illumination condition for NTT [23]. Background noise was kept at a minimum during the test. All experimental parameters were monitored closely and kept as consistent as possible throughout the study. The part of the tank filled with water (11.5-cm height) was virtually divided into top, center, and bottom of equal heights (approximately 3.8 cm per segment) for the analyses. The average time spent at the top and bottom portions of the tank was analyzed to determine the anxiety-like behavior of fish. The average total distance traveled and freezing time (as defined by a complete cessation of movement except for gills and eyes [24]) were calculated to determine the effects of the compounds on the general behavior of fish. The analyses were conducted for 1-min time bins. To account for any day-to-day variability in fish behavior, fish treated with vehicle were tested on the same day as the compounds. Any fish that stayed immobile for longer than 200 s out of a total of 5-min test period were considered as an outlier, because it was generally > 2 standard deviations away from the mean, and thus, excluded from the analyses. Three fish from vehicle control, one fish from 10 mg/L anatabine, and 3 fish from 100 mg/L buspirone were removed from the final analysis, but these changes did not significantly alter the statistical results. A minimum of 12 fish (6 females and 6 males) per condition were used for the study. The actual sample size per condition is indicated in Online Resource 1. For some compounds, the study was repeated to confirm reproducibility and the results were combined together. The experimenters were blind to the test conditions. The NTT was validated with buspirone, a clinical anxiolytic drug, as previously described [6].

The test concentrations were determined by first testing the compounds at 30 mg/L. If the fish tolerated the concentration for 20 min (as determined by lack of abnormal behaviors, such as tail or body tremors or floating at the surface of the water), then higher concentrations were tested. If not, the concentration was reduced until no obvious signs of tolerability problems were observed. The test concentrations for the NTT were as follows: nicotine (0.3, 1, and 3 mg/L; equivalent to 2, 6, and 19 µM), cotinine (30, 100, and 300 mg/L; equivalent to 171, 568, and 1705 µM), anatabine (0.3, 1, 3, and 10 mg/L, equivalent to 2, 6, 19, and 63 µM), methylanatabine (1, 3, and 10 mg/L; equivalent to 4, 12, and 40 µM), anabasine (0.3, 1, and 3 mg/L; equivalent to 2, 6, and 19 µM), nornicotine (3, 10, and 30 mg/L; equivalent to 20, 68, and 203 µM), metanicotine oxalate (30, 100, and 300 mg/L; equivalent to 119, 397, and 1190 µM), AZD1446 (30, 100, and 300 mg/L; equivalent to 124, 415, and 1245 µM), acetylcholine (30, 100, and 300 mg/L; equivalent to 132, 441, and 1322 µM), and buspirone (10, 30, and 100 mg/L; equivalent to 26, 78, and 259 µM). The concentrations were calculated based on the free base molecular weight. Buspirone, a clinical anxiolytic drug, was included as a positive control to confirm the validity of the NTT.

### Brain dissection

Four fish (2 males and 2 females) per compound were used to determine the brain bioavailability of the compounds. Immediately after the 20-min compound treatment, the zebrafish were rinsed briefly to remove excessive compound on their body and terminated with 250 mg/L tricaine (CAS No. 886-86-2; Sigma). The fish were decapitated at the level of the gills using a surgical knife. The head was turned dorsal side down, and soft tissue was removed from the ventral side of the skull until the base of the skull was exposed. The skull was broken open and the bone from the ventral side of the brain was removed. The brain was then placed in a microcentrifuge tube, weighed, snap-frozen in liquid nitrogen, and stored at – 80 ºC until the analysis.

### Bioavailability assay

Briefly, on the day of analysis, the brain samples were defrosted, resuspended in a methanol solution (2:1 [v/v] methanol:MilliQ water), and homogenized with vigorous agitation and ultrasonication (5 min each). The homogenate was centrifuged at 15,000 × rpm for 10 min, and the supernatant was analyzed using a UPLC-Q Exactive Orbitrap-HRMS system (Thermo Fisher Scientific™, Bremen, Germany). Chromatographic separation was achieved on a Synergi™ 4-µm Hydro-RP 80 Å, L.C. Column (250 × 4.6 mm; Phenomenex Inc., Torrance, CA, USA) with 0.1% formic acid in water (mobile phase A) and 0.1% formic acid in acetonitrile (mobile phase B). A gradient method at a 500 µL/min flow rate was applied as follows: (1) 5% B for 1 min, and (2) increase to 95% B over 7 min and hold for 2 min. The injection volume was 5 μL. The mass spectrometer was operated in electrospray positive mode (ESI, Thermo Fisher Scientific), while data acquisition was performed using the parallel reaction monitoring (PRM) and full scan modes. The source settings were set as follows: sheath gas flow rate = 60 psi; aux gas flow rate = 20 arbitrary units; spray voltage = 3.5 kV; capillary temperature = 280 °C; and sweep gas flow rate = 1. The full scan mode parameters were set as follows: resolution = 35,000 FWHM at 200 m/z; AGC target = 1E6; maximum injection time = 110 ms; and scan range = 100–350 m/z. The chromatographic and Orbitrap MS parameters for PRM analysis were the same as those in the full scan mode, except for: AGC target = 2E5; maximum IT = 60 ms; and resolution = 17,500 FWHM at 200 m/z. The XCalibur™ v4.0.27.19 software (Thermo Fisher Scientific) and TraceFinder™ v4.1 Forensic (Thermo Fisher Scientific, San José, CA, USA) were used for system control and data processing, respectively. The Q Exactive 2.8 SP 1 software (Thermo Fisher Scientific) was used to control the mass spectrometer.

### In vitro nAChR functional assay

Electrophysiological responses were recorded using an automated patch-clamp Patchliner Octo^®^ system (Nanion Technologies, Munich, Germany) equipped with two EPC-10 Quadro patch-clamp amplifiers (HEKA Elektronik, Lambrecht, Germany) as described previously [2]. Chinese hamster ovarian (CHO) cells stably expressing human α4β2 nAChRs (Charles River Laboratories, Wilmington, MA, USA) were used. All experiments were performed at room temperature (24 °C) and repeated at least three times. Data were analyzed using Patchmaster software (HEKA Elektronik). Offline data analysis was performed in Apache OpenOffice™ (v4.1.2; Microsoft, Redmond, WA, USA). Igor Pro software (v6.2.2.2; WaveMetrics, Lake Oswego, OR, USA) was used to determine EC_50_ values. The efficacy of the compounds was calculated by first normalizing the current induced by each compound by the internal acetylcholine control. These values were then expressed as a percentage of maximum receptor activation by nicotine. The average values were then fitted to the Hill equation: $I = {\text{Baseline}} + \left( {X^{ \wedge } nH} \right)(\text{I} _{{\max }} - {\text{Baseline}})/\left( {X^{ \wedge } nH + EC50^{ \wedge } nH} \right),$where *I* is the current response, *Baseline* is the minimal current response, *X* is the agonist concentration, *nH* is the Hill coefficient, *I*_*max*_ is the maximal current, and *EC*_*50*_ is the agonist concentration producing half-maximal activation. Data are presented as mean ± SD.

### In vitro molecular target profiling

One hundred and sixty-five molecular targets were selected based on various references and databases. Majority of targets were selected using SuperPred database as a guide for known and predicted targets of the three compounds [42]. SuperPred is a publicly accessible database that provides both experimentally reported drug–target interactions (DTIs) and predicted DTIs derived by a molecular similarity approach, covering a total of 665,000 DTIs connecting 31,000 compounds and 1800 targets [42]. This database was chosen because of its comprehensive coverage for nicotine, anatabine, and cotinine compared to other databases [17]. Additional targets were included based on previous in-house proteomics and behavioral profiling/drug classification investigations, the abuse potential guidelines published by the United States Food and Drug Administration in 2017 [65], and preclinical drug safety screening guidelines [9, 68]. Combining the results of these resources, nicotine, cotinine, and anatabine were tested in technical duplicates against 175 assays including, for example, 86 GPCRs, 23 ion channels, 7 transporters, 15 kinases, and 35 other enzymes.

All binding and functional assays for molecular target characterization were conducted by Eurofins Cerep SA (Celle-Lévescault, France) and Eurofins Panlabs Discovery Services Taiwan, Ltd. (New Taipei City, Taiwan) using their standard in vitro binding and functional assays (Online Source 2). The radioligand displacement binding assays employed the gold-standard filtration method using membrane preparations from stable cell lines expressing human or rodent target proteins to determine the interaction of the compounds with specific receptors, channels, and transporters. For this purpose, the competitive binding of test compounds against a [^125^I]-, [^3^H]-, or [^35^S]-labeled agonist and/or antagonist was determined. The specific list of radiolabeled ligands and experimental conditions are summarized in Online Source 2. A single concentration of each compound (10 µM in 0.1% DMSO) was used for the screen. Compounds that showed an effect greater than 50% was considered significant. Negative values were considered to be an artifact arising from, for example, compounds interfering with the assay readout.

### Molecular docking

The crystal structure of human nAChR α4β2 (PDB ID 5KXI) was retrieved from the Protein Data Bank [7]. The structure was loaded onto Molecular Operating Environment (MOE) software (2019.01; Chemical Computing Group ULC, Montreal, QC, Canada) and prepared for molecular docking using the “structure preparation” feature of the software. The AMBER14 force field was selected to calculate the interaction energies of the ligand–protein binding.

The chemical structures of nicotine, cotinine, anatabine and methylanatabine were retrieved from PubChem [27] and the dominant protonation state at pH 7.4 was determined using the ChemAxon Major Microspecies Plugin (ChemAxon, Budapest, Hungary). The agonist-binding site for docking simulation of the human nAChR α4β2 was identified around the co-crystallized nicotine ligand [41]. Nicotine, anatabine and methylanatabine were docked in the protonated form and cotinine in the neutral form according to their dominant protonation form at pH 7.4. The molecular docking was conducted in a flexible manner (“induced fit”). The “triangle matcher” function was selected as the placement method and the London ΔG as the scoring function, which estimates the free energy of binding of the ligand from a pose, knowing that the lowest ΔG values correspond to the highest binding affinity. The best poses were refined and rescored using the Generalized Born Volume Integral/Weighted Surface Area (GBVI/WSA) ΔG score and the binding energy value was extracted from the best pose. The GBVI/WSA ΔG is a force field-based scoring function, which estimates the free energy of binding of the ligand from a given pose [12]. Molecular interactions between protein–ligand complexes were analyzed.

### In silico blood–brain barrier (BBB) permeability prediction

The Ligand Express^®^ (Cyclica; Toronto, Ontario, Canada) and admetSAR platforms were used to predict BBB permeability of the compounds. Ligand Express^®^ is a cloud-based platform that screens small-molecule drugs against repositories of structurally characterized proteins or ‘proteomes’ to determine polypharmacological profiles. In terms of BBB prediction, the system implements a classification model based on machine-learning methods using a compiled BBB dataset of 1335 BBB-permeable and 360 BBB-impermeable compounds. The Anatomical Therapeutic Chemical Classification is used to filter out compounds that have an ambiguous status regarding their passage through the BBB or were not strictly CNS-active. In addition, 45 molecules that are known to cross the BBB and 91 P-gp substrates on the BBB-impermeable set were included [1, 55, 56].

The admetSAR (v2.0) server was developed as a comprehensive source and free tool for the in silico prediction of chemical absorption, distribution, metabolism, excretion, and toxicity (ADMET) properties based on structure–activity relationships (SAR) [11, 71]. More than 40 predictive models are implemented in admetSAR for in silico filtering of new chemical ADMET properties. These models are trained by state-of-the-art machine-learning methods. The BBB model was developed using a similar dataset used by Ligand Express^®^, derived mainly from the work of Shen et al. which included 1839 compounds (1438 BBB-permeable and 401 BBB-impermeable compounds) [56]. Because both of these platforms gave almost identical BBB penetration probability values for all compounds, only the results from Ligand Express^®^ are described in the result. Values equal to or close to 1 indicate compounds with a high probability of BBB penetration.

Lastly, the Biovia Pipeline Pilot ADME-Tox Blood Brain Barrier Model (Dassault Systèmes, Vélizy-Villacoublay, France) was used to predict the BBB penetration of a molecule, defined as the ratio of concentrations (brain concentration/blood concentration) after oral administration, and to report the predicted penetration as well as a classification of penetration level. The model combines a confidence ellipse, in the polar surface area and LogP descriptor space, derived from over 800 orally administered compounds classified as CNS therapeutics with a robust regression model based on 120 compounds with measured penetration to predict Log(Brain Blood (BB)) penetration values for those molecules falling within the confidence ellipse [15]. The model predicts the BB permeation level based on the categories “very high” (BB ratio > 5:1), “high” (between 1:1 and 5:1), “medium” (between 0.3:1 and 1:1), “low” (< 0.3:1), and “undefined” (outside the 99% confidence range ellipse). This translates for the regression model prediction values of Log(BB) > 0.7 for “very high”, 0 < Log(BB) < 0.7 for “high”, -0.52 < Log(BB) < 0 for “medium”, and Log(BB) < -0.52 for “low”. No prediction is made for compounds outside the 95% confidence ellipsoids.

### Statistics

Two-way repeated measures analysis of variance (ANOVA) with Dunnett post hoc was used for the 1-min binned analysis of the zebrafish NTT data. Treatment was one factor, and time was the second factor. Sex was not included, because the initial assessment of the dataset indicated no sex difference. Thus, male and female datasets were combined for the final analyses. The epsilon values of sphericity were never higher than 1.0 and all data passed the Shapiro–Wilk normality test. The EC_50_ values were determined by applying the nonlinear regression analysis. All analyses were conducted using GraphPad Prism v8.2.1 (GraphPad Software, San Diego, CA, USA).

## Results

### Functional activities on α4β2 nAChR

**Fig. 1:**
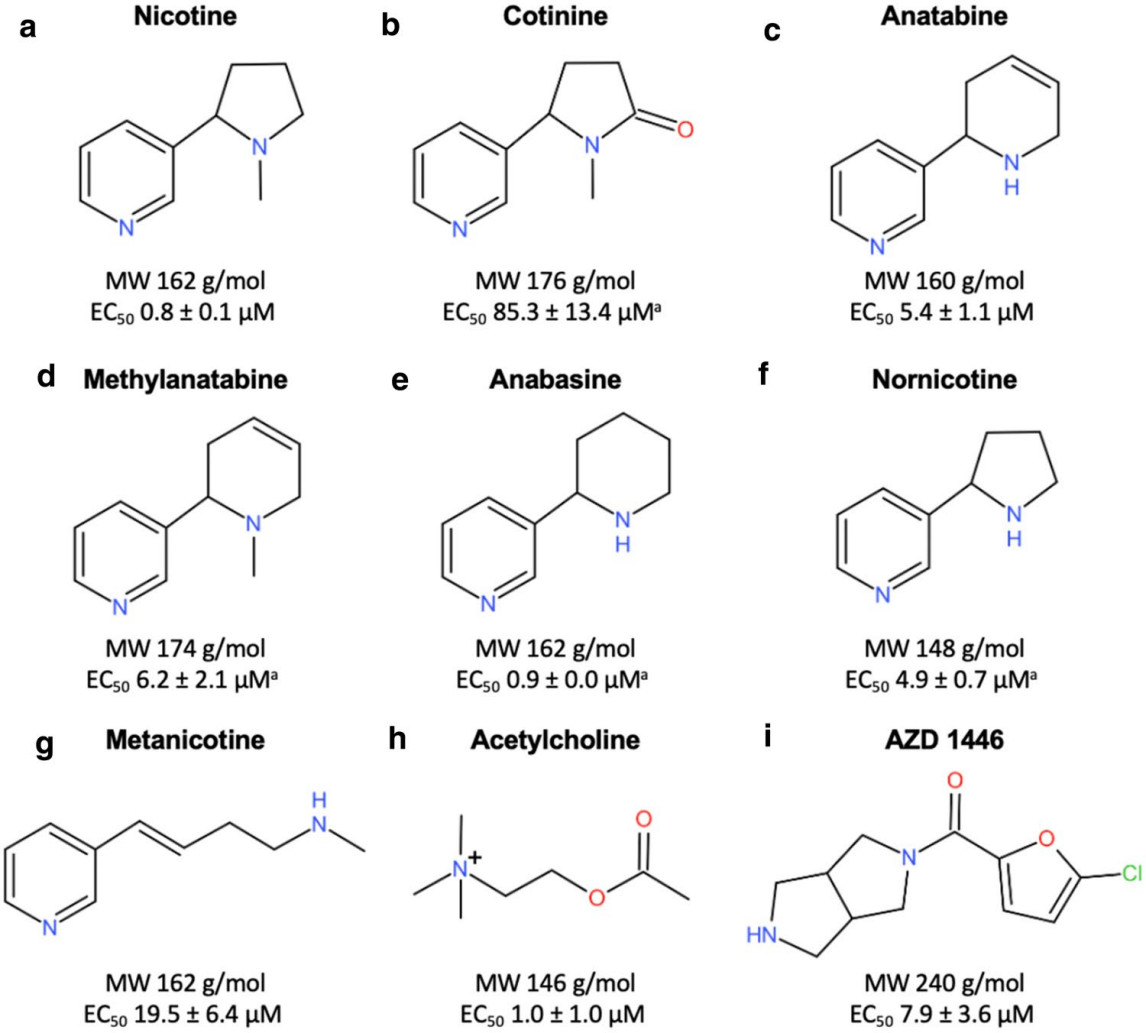
Summary of compounds. The chemical structure, molecular weight (MW), and α4β2 nAChR EC_50_ are presented for all test compounds: **a** nicotine, **b** cotinine, **c** anatabine, **d** methylanatabine, **e** anabasine, **f** nornicotine, **g** metanicotine, **h** acetylcholine, and **i** AZD1446. The EC_50_ values are presented in mean ± SEM. ^a^Potential partial agonists are cotinine (59%), methylanatabine (26%), anabasine (7%), nornicotine (44%) (Online Source 3). The percentages in the parentheses are calculated based on the maximum receptor activation by nicotine as 100%

**Fig. 2:**
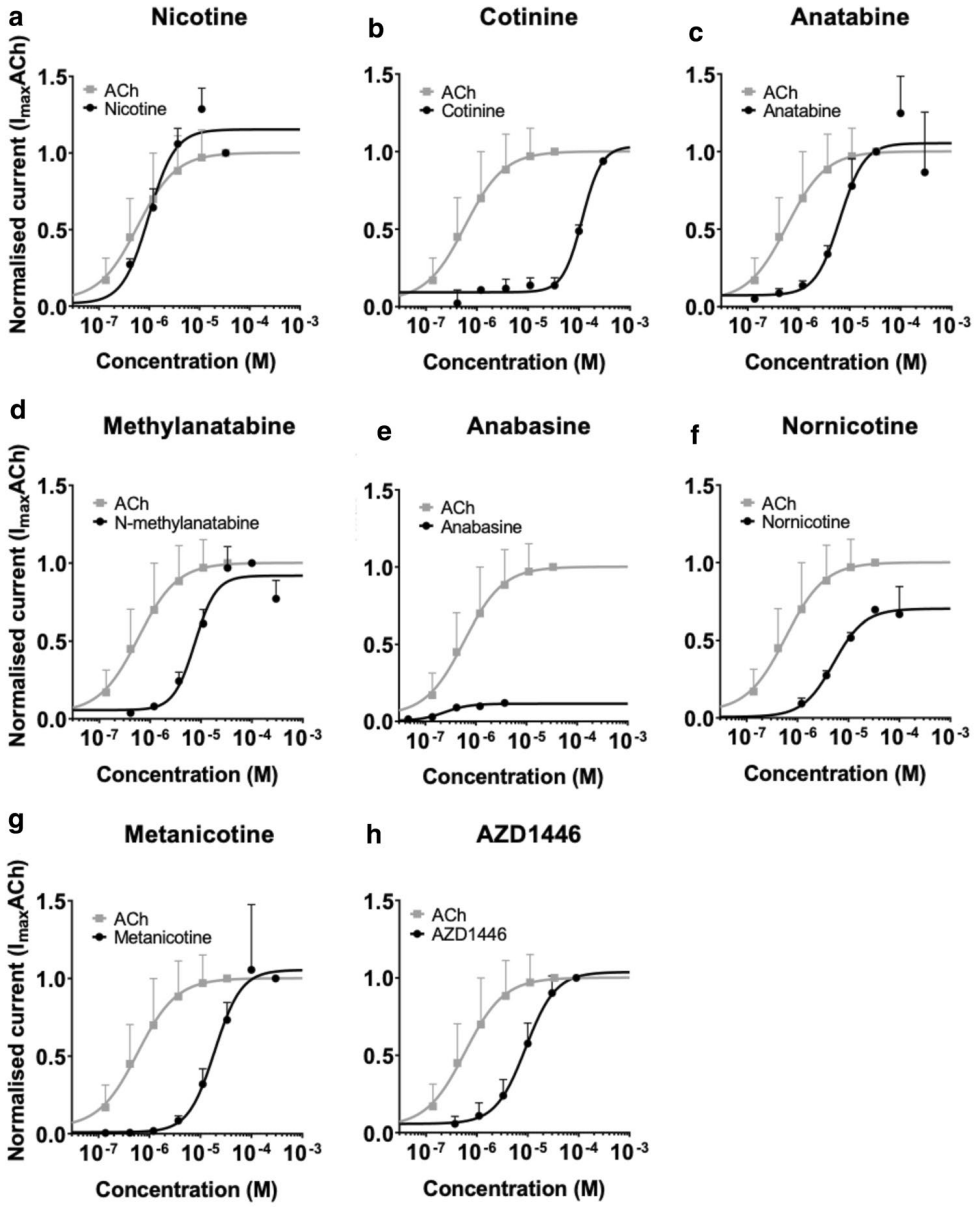
α4β2 nAChR activation by alkaloids in vitro. Dose–response curves for α4β2 nAChR activation by respective alkaloids in vitro are presented for **a** nicotine, **b** cotinine, **c** anatabine, **d** methylanatabine, **e** anabasine, **f** nornicotine, **g** metanicotine, and **h** AZD1446 in black lines. The grey lines represent the fits to the Hill equation for acetylcholine. The average values were fit to the Hill equation. The EC_50_ values are presented in Fig. 1. The averag normalized current response is plotted as a function of the maximal current to acetylcholine (I_maxACh_) (*n* = 3–11). The acetylcholine curve was replotted for each graph. Data are shown as mean ± SD

To confirm and understand the different potencies of alkaloids and reference compounds, their functional activities on α4β2 nAChRs were confirmed using α4β2 nAChR-overexpressing CHO cells. Among them, nicotine, anatabine, methylanatabine, anabasine, nornicotine, acetylcholine, and AZD1446 had EC_50_ < 8 µM. Cotinine had relatively low potency in activating α4β2 nAChRs, with EC_50_ of 85.3 ± 13.4 (Figs. 1 and 2). These results confirmed that all alkaloids tested in the zebrafish NTT can activate α4β2 nAChR albeit at different potencies. Interestingly, we found that not all alkaloids fully activated α4β2 nAChR (Online Resource 3). For example, compared to nicotine, anabasine only partially activated α4β2 nAChR (7% of maximum activation by nicotine) despite of having a very similar potency as nicotine (EC_50_ = 0.8 ± 0.1 µM for nicotine vs. 0.9 ± 0.0 µM for anabasine). Similarly, cotinine (59%), methylanatabine (26%), and nornicotine (44%) also did not induce full activation of α4β2 nAChR. It is worth noting that independent functional assays were conducted for each compound, and, thus, the possible roles of the compounds as non-competitive or silent agonists or allosteric modulators were not assessed.

### Effects of compounds on zebrafish NTT response

**Fig. 3:**
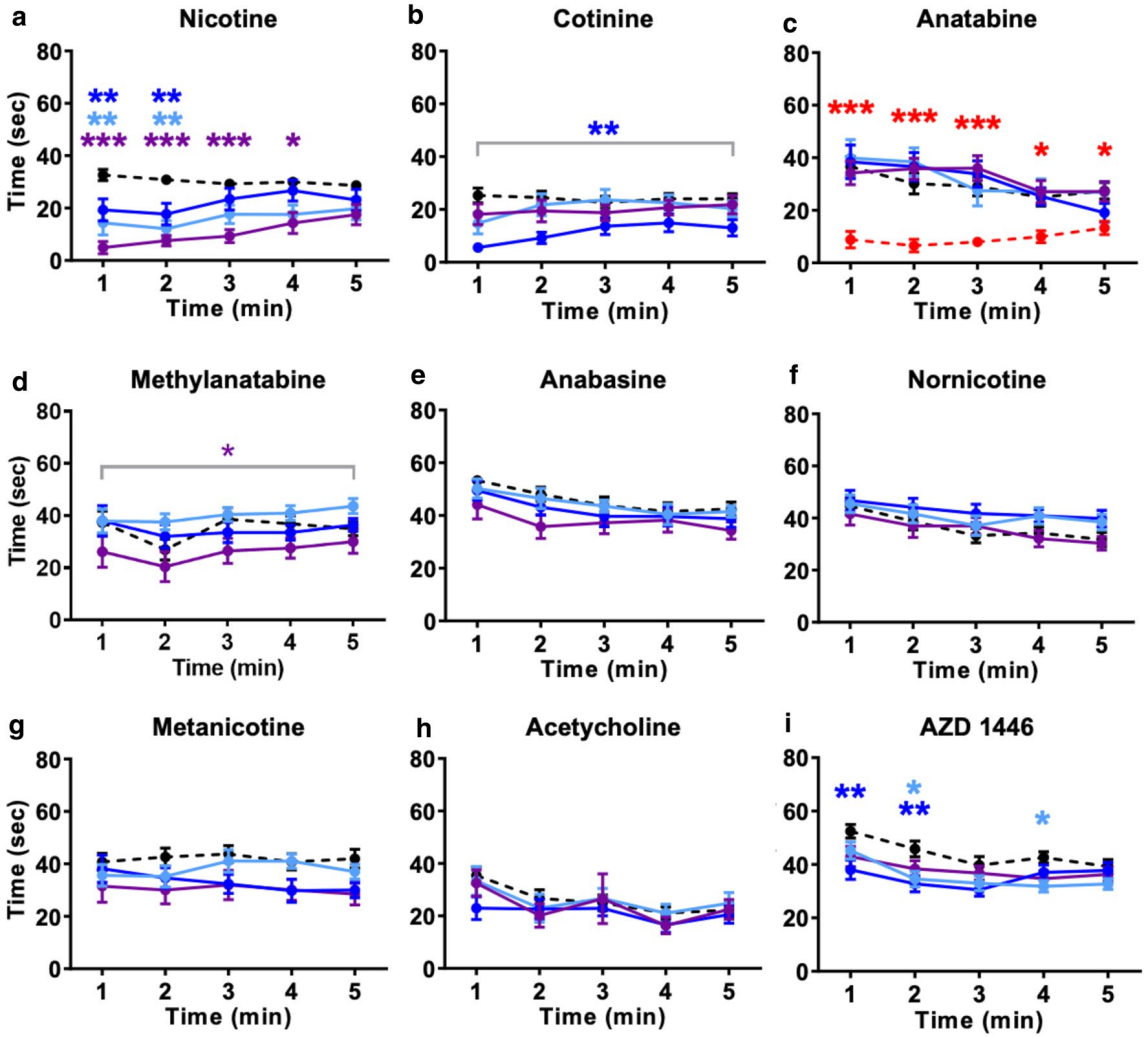
Time spent at the bottom of the tank during NTT. Time spent at the bottom of the tank during NTT is presented for **a** nicotine (0.3, 1, and 3 mg/L), **b** cotinine (30, 100, and 300 mg/L), **c** anatabine (0.3, 1, 3, and 10 mg/L), **d** methylanatabine (1, 3, and 10 mg/L), **e** anabasine (0.3, 1, and 3 mg/L), **f** nornicotine (3, 10, and 30 mg/L), **g** metanicotine (30, 100, and 300 mg/L), **h** acetylcholine (30, 100, and 300 mg/L), and **i** AZD1446 (30, 100, and 300 mg/L). Black dashed lines = control; light blue = lowest concentration; blue = middle concentration; purple = highest concentration. For anatabine only, red is the highest concentration. *p < 0.05; **p < 0.01; ***p < 0.001 compared to the vehicle control. Data are expressed as mean ± SEM

Zebrafish were placed in an NTT tank immediately after freely swimming in water containing one of the seven alkaloids (nicotine, cotinine, anatabine, anabasine, metanicotine, nornicotine, or methylanatabine) or reference compounds (acetylcholine, AZD1446, or buspirone) for 20 min. All three concentrations of nicotine (0.3, 1, and 3 mg/L) reduced the time spent at the bottom of the tank during the first 2 min of the test (*p* < 0.01) (Fig. 3a; treatment x time effect: F(12, 524) = 2.512, *p* = 0.0032). Fish exposed to the highest concentration of nicotine continued to spend less time at the bottom of the tank during the 3rd and 4th min of the test (*p* < 0.001 at 3 min; *p* < 0.01 at 4 min). One hundred milligram per liter cotinine reduced the overall time spent at the bottom (*p* < 0.01), but not at 30 or 300 mg/L (Fig. 3b; treatment effect: F(3, 92) = 3.691, p = 0.0147; no significant effect of the treatment x time). Anatabine significantly decreased the time spent at the bottom (*p* < 0.001 upto 3 min; *p* < 0.05 during the last 2 min) only at the highest concentration (10 mg/L) tested (Fig. 3c; treatment x time effect: F(16, 316) = 2.568, *p* = 0.0009). The anxiolytic-like effect of methylanatabine was also only observed at the highest concentration tested (*p* < 0.05) (Fig. 3d; treatment effect: F(3, 44) = 4.199, *p* = 0.0107). The observed reductions in the time spent at the bottom of the tank for respective compounds were also clearly reflected by the corresponding increase in the time spent at the top of the tank (Online Resource 4). Anabasine, nornicotine, and metanicotine had no significant effect (Fig. 3e–g and Online Resource 3e–g).

Acetylcholine, an endogenous nAChR ligand, had no effect on the time spent on the bottom or top of the tank (Fig. 3h and Online Resource 4 h). A hundred milligram per liter AZD1446, a α4β2 nAChR reference compound, decreased the time spent at the bottom for the first 2 min (*p* < 0.01) (Fig. 3i; treatment x time effect: F(12, 360) = 1.797, *p* = 0.0471). Thirty milligram per liter AZD1446 decreased the time spent at the bottom at 2 and 4 min (*p* < 0.05). AZD1446 also increased the time spent at the top at these concentrations (Online Resource 4i; treatment effect: F(3, 90) = 3.158, *p* = 0.0285).

**Fig. 4:**
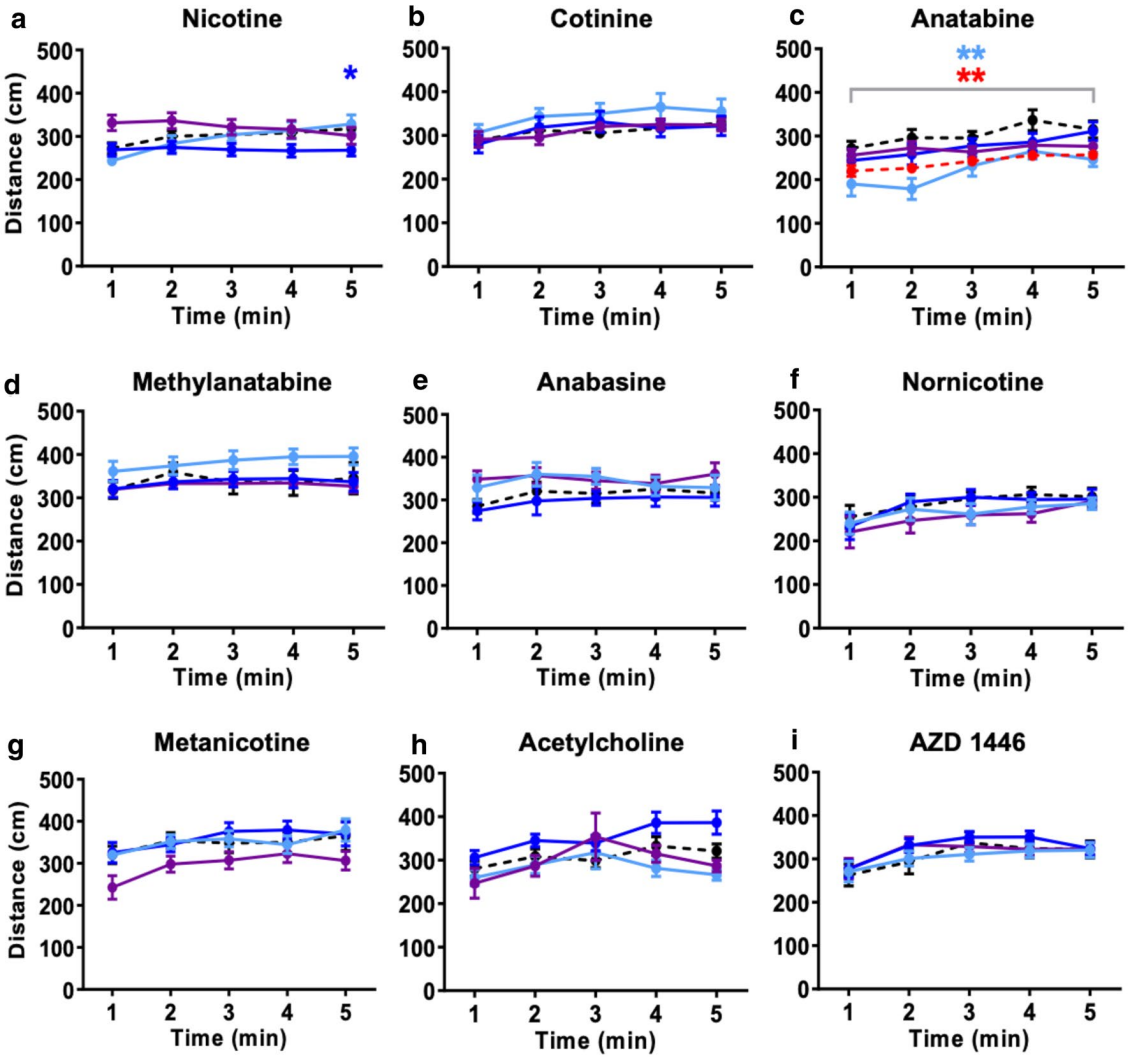
Effects of compounds on total distance traveled. Total distance traveled in the entire tank during the NTT is presented for **a** nicotine (0.3, 1, and 3 mg/L), **b** cotinine (30, 100, and 300 mg/L), **c** anatabine (0.3, 1, 3, and 10 mg/L), **d** methylanatabine (1, 3, and 10 mg/L), **e** anabasine (0.3, 1, and 3 mg/L), **f** nornicotine (3, 10, and 30 mg/L), **g** metanicotine (30, 100, and 300 mg/L), **h** acetylcholine (30, 100, and 300 mg/L), and **i** AZD1446 (30, 100, and 300 mg/L). Black dashed lines = control; light blue = lowest concentration; blue = middle concentration; purple = highest concentration. For anatabine only, red is the highest concentration. *p < 0.05; **p < 0.01. Data are expressed as mean ± SEM

The general activity, measured by total distance traveled and freezing time, was not greatly affected by these compounds except for anatabine (Fig. 4 and Online Resource 5). Anatabine induced a slight but significant reduction in the total distance traveled at the lowest (0.3 mg/L) and highest (10 mg/L) concentrations (*p* < 0.01 for both) (Fig. 4c; treatment effect: F(4, 79) = 4.153, *p* = 0.0042). Similarly, 0.3 mg/L anatabine increased the freezing time during the first 3 min of the test (*p* < 0.001 for 1st 2 min, *p* < 0.05 for 3rd min) (Online Resource 5c; treatment × time effect: F(16, 316) = 3.059, *p* < 0.0001). These observed changes in general activity did not seem to reflect the anxiolytic-like effects, because only 10 mg/L anatabine decreased the time spent at the bottom.

To confirm the validity of the NTT, a clinical anxiolytic drug buspirone was tested in parallel. All concentrations of buspirone either reduced the time spent on the bottom or increased the time spent on the top at multiple time points (Online Resource 6a & b; treatment x time effect for the bottom: F(12, 280) = 4.030, *p* < 0.0001; for the top: F(12, 280) = 3.838, *p* < 0.0001). Thirty milligram per liter buspirone, in particular, induced consistent anxiolytic-like effect across all time points (*p* < 0.001). When the general activity was analyzed, 100 mg/L buspirone reduced the total distance traveled at 1, 4, and 5 min (p < 0.01) and increased the freezing response at 1, 2, and 5 min (*p* < 0.001 at 1 min; p < 0.05 at 2 and 5 min) (Online Resource 6c & d; treatment × time effect for distance traveled: F(12, 280) = 3.693, *p* < 0.0001; treatment × time effect for freezing: F(12, 280) = 3.311, *p* = 0.0002). These observed changes in general activity did not seem to reflect the anxiolytic-like effects observed for buspirone, because all concentrations induced anxiolytic-like effects instead of just those concentrations affecting the general activity parameters.

### BBB permeability and brain bioavailability

To understand whether the alkaloids and reference compounds have good BBB permeability, two qualitative classification models for predicting the probability of BBB penetration (Ligand Express^®^ and admetSAR) and one quantitative regression model for predicting the LogBB compound penetration values when taken orally (Biovia ADMET) were used. The Ligand Express^®^ predicted all compounds to have good BBB penetrability as indicated by a probability close to 1 (Table 1). The admetSAR model provided more granular differences among the compounds. The predicted LogBB values by the Biovia Pipeline Pilot ADME-Tox BBB  model for most compounds were in the medium range (– 0.52 to 0), with the exception of cotinine and AZD1446, which were in the low range (< – 0.52), and methylanatabine, which was in a high range (> 0) (Table 1). According to this model, acetylcholine was predicted as outside the confidence range of the model.

**Table 1:** In silico BBB penetration prediction and bioavailability of compounds in zebrafish brains

Compounds	BBB ligand express^®^	Log (BB) Biovia ADMET	Relative brain level per 1 mg/L compound^b, c^ (ng/mg tissue)
Nicotine	1.000	– 0.001 (Med)	0.70 ± 0.14 (0.01 ± 0.01 cotinine)
Cotinine	1.000	– 0.567 (Low)	0.02 ± 0.01
Anatabine	0.987	– 0.271 (Med)	0.44 ± 0.15
Methylanatabine	0.991	0.044 (High)	1.39 ± 0.56
Anabasine	0.994	– 0.176 (Med)	0.23 ± 0.07
Nornicotine	0.988	– 0.317 (Med)	0.07 ± 0.01
Metanicotine	0.942	– 0.224 (Med)	0.07 ± 0.01
acetylcholine	0.996	No value^a^	0.02 ± 0.01
AZD1446	0.994	– 0.803 (Low)	No value^d^
Varenicline	0.994	– 0.392 (Med)	0.45 ± 0.16

^a^Outside the confidence range of the model

^b^Data are presented as mean ± SEM

^c^Relative compound level in the brain per 1 mg/L compound exposure was calculated by assuming a linear relationship between compound concentration and blood–brain barrier penetration

^d^Below the detection level of 0.1–0.2 ng/mL

To confirm the predicted BBB penetration values, the brain bioavailability of the compounds was quantified from the fish brains. Nicotine, anatabine, and methylanatabine, were found at relatively expected concentrations close to 1 ng/mg brain tissue per 1 mg/L compound exposure (Table 1). Cotinine, nornicotine, metanicotine, and acetylcholine concentrations were relatively low, all measuring less than 0.1 ng/mg brain tissue per 1 mg/L compound exposure. The concentration of AZD1446 could not be detected under the assay condition used in this study. Overall, the pattern of the relative brain concentrations correlated well with the BBB penetration prediction obtained using the Biovia ADME-Tox BBB model.

### In vitro α4β2 nAChR-binding potencies of alkaloids

**Fig. 5:**
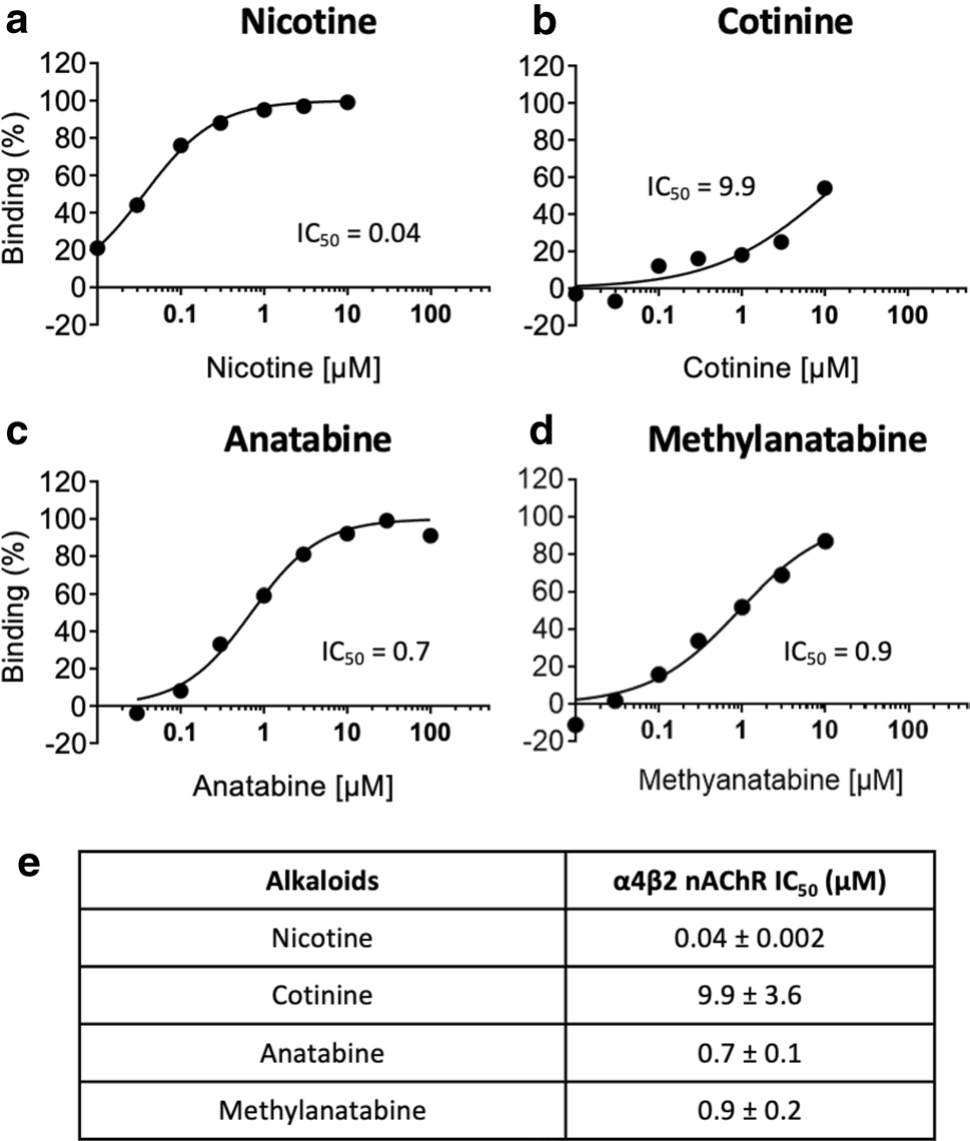
α4β2 nAChR binding by alkaloids in vitro. Dose–response curves for α4β2 nAChR binding by respective alkaloids in vitro are presented for **a** nicotine, **b** cotinine, **c** anatabine, and **d** methylanatabine. The average EC_50_ values are included in each figure and summarized in panel (**e**). *n* = 3. Data are shown as mean ± SD

To further investigate the receptor pharmacology of nicotine, cotinine, anatabine, and methylanatabine, we tested the binding affinity of these alkaloids to α4β2 nAChRs in vitro. In agreement with the receptor activity, nicotine showed the strongest binding affinity towards α4β2 nAChRs (IC_50_ = 0.04 ± 0.002 µM), followed by anatabine and methylanatabine (IC_50_ = 0.7 ± 0.1 and 0.9 ± 0.2 µM, respectively), then cotinine (IC_50_ = 9.9 ± 3.6 µM, respectively) (Fig. 5).

Furthermore, to understand potential off-target effects of the compounds, we selected 175 in vitro binding and enzymatic assays to determine the molecular target specificity of nicotine, cotinine, and anatabine based on the database and previous studies. Methylanatabine was not analyzed due to its close similarly to the other three alkaloids. The result indicated that nicotine, cotinine, and anatabine showed specific binding to α4β2 and muscle-type nAChR and did not bind or regulate the activities of other molecular targets in vitro (Online Source 7).

### In silico molecular docking prediction

**Fig. 6:**
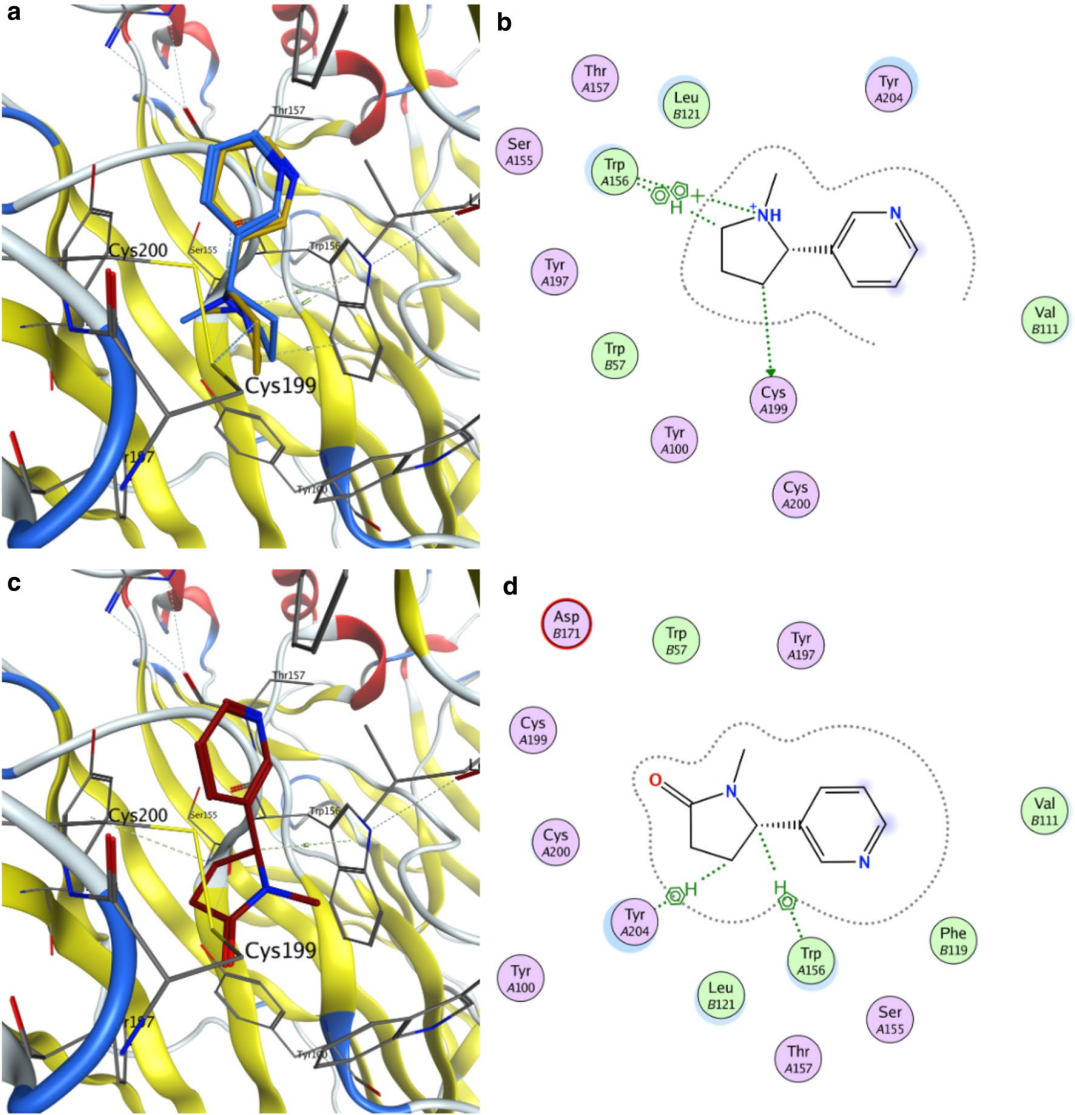
Docking positions of nicotine and cotinine at the α4β2 nAChR agonist-binding site. Docking positions of **a** nicotine (carbon atoms in blue) and **c** cotinine (carbon atoms in red) at the α4β2 nAChR agonist-binding site are shown. The nicotine pose docked in a similar position as the crystalized pose (carbon atoms in gold), with a binding energy of − 6.3 kcal/mol. The 2D interaction schemes show that **b** nicotine interacts with Trp156 and Cys199 and **d** cotinine with Trp156 and Tyr204. The interactions are shown in dashed lines between nicotine or cotinine and the receptor residues. Pink circles represent polar residues. Green circles represent hydrophobic residues

Molecular docking was carried out using the human nAChR α4β2 crystal structure. Nicotine, cotinine, anatabine, and methylanatabine were docked in the binding pocket described as the agonist-binding site of nicotine in the retrieved crystal structure [41]. Figure 6a shows the nicotine pose generated by docking (carbon atoms in blue) and its comparison with the crystalized pose (carbon atoms in gold). The docking algorithm placed nicotine successfully in the agonist-binding site, which closely overlapped with the reported crystal structure complex. The model revealed an aromatic interaction between the protonated pyrrolidine ring of nicotine and Trp156 and a polar interaction with Cys199. The obtained binding affinity energy (ΔG =  − 6.3 kcal/mol) was in agreement with that reported by Schapira, et al. (ΔG =  − 6.02 kcal/mol) [54].

**Fig. 7:**
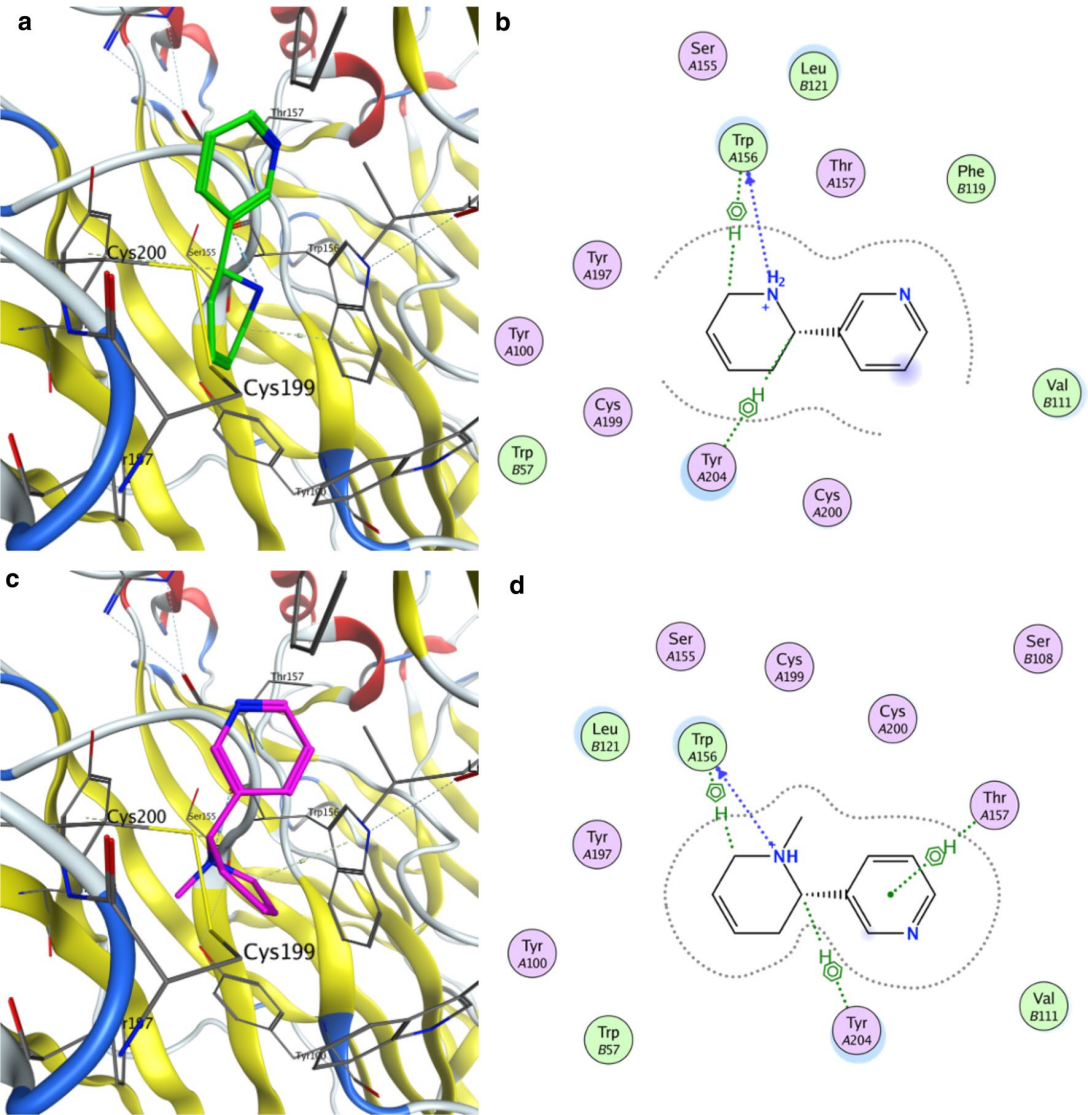
Docking positions of anatabine and methylanatabine at the α4β2 nAChR agonist-binding site. Docking positions of **a** anatabine (carbon atoms in green) and **c** methylanatabine (carbon atoms in pink) at the α4β2 nAChR agonist-binding site are shown. The 2D interaction schemes show that **b** anatabine interacts with Trp156 and Tyr204 and **d** methylanatabine with Trp156, Tyr204, and Thr157. The interactions are shown in dashed lines between nicotine or cotinine and the receptor residues. Pink circles represent polar residues. Green circles represent hydrophobic residues.

Similarly, the best docking poses for cotinine, anatabine, and methylanatabine were analyzed (Figs. 6b and 7). The docked positions of cotinine, anatabine, and methylanatabine were similar to the nicotine position. The pyridine ring of all alkaloids was positioned in the same region of the binding pocket with the nitrogen pointing in the same direction with the exception of methylanatabine. This difference resulted in an aromatic interaction of the pyridine ring with Thr157, which was not observed in the other alkaloids. Similar to nicotine, the pyrrolidine ring of cotinine and the 1,2,3,6-tetrahydropyridine rings of anatabine and methylanatabine showed aromatic interactions with Trp156. In the case of nicotine, anatabine and methylanatabine, a polar interaction was also observed between this residue and the protonated nitrogen of the rings. The interaction observed between Cys199 and the nicotine pyrrolidine ring was not observed for the other three alkaloids. Instead, this interaction was replaced by an interaction with Tyr204, which may explain the lower predicted affinity of these alkaloids compared to nicotine.

The predicted binding affinity energy of the four alkaloids using the molecular docking model was the highest for nicotine (– 6.31 kcal/mol), followed by anatabine and methylanatabine (– 6.03 and 6.23 kcal/mol, respectively), then cotinine (– 5.71 kcal/mol) (Table 2). The predicted affinities were very similar to the EC_50_ and IC_50_ values obtained from the in vitro nAChR α4β2 functional and binding assays, respectively (Table 2), where nicotine was shown to be the most potent alkaloid.

**Table 2:** Comparison of experimental EC_50_ and IC_50_ values with in silico docking prediction

Compounds	α4β2 nAChR EC_50_ (μM)	α4β2 nAChR pEC_50_ (M)^a^	α4β2 nAChR IC_50_ (μM)	α4β2 nAChR pIC_50_ (M)^a^	α4β2 nAChR-binding affinity (kcal/mol)
Nicotine	0.8	6.10	0.04	7.40	– 6.31
Cotinine	85.3	4.07	9.9	5.00	– 5.71
Anatabine	5.4	5.27	0.7	6.15	– 6.03
Methylanatabine	6.2	5.21	0.9	6.05	– 6.23

^a^ EC_50_ and IC_50_ values were converted to log10 values (pEC_50_ and pIC_50_, respectively) to compare to the binding affinity determined by the docking model

## Discussion

Zebrafish have become a model vertebrate organism for studying complex molecular and cellular interactions in vivo over the last few decades [16, 49, 58, 60]. Many publications now support the suitability of zebrafish to model various aspects of anxiety-related states [26, 30, 58]. In fact, zebrafish NTT has been validated using several clinical anxiolytic compounds, including buspirone, fluoxetine, diazepam, chlordiazepoxide, and tranylcypromine [5, 6, 10, 14, 18, 22, 25, 26, 29, 30, 32, 36, 37, 39, 44, 50, 53, 57–59, 66] and has also shown to be sensitive to nicotine and other nicotinic ligands [5, 28, 32, 33, 53, 67]. In this study, we investigated the effects of nicotine and six additional nAChR-activating alkaloids on the NTT. Of the alkaloids tested, nicotine was the most potent compound to reduce the anxiety-like behavior in zebrafish, showing an efficacy even at 0.3 mg/L. The two metabolites of nicotine—cotinine and nornicotine—could not account for the anxiolytic-like effect of nicotine found in this study, because cotinine induced an effect at a concentration > 300-fold higher than nicotine and nornicotine had no effect. Furthermore, the level of cotinine in the zebrafish brain after nicotine treatment was 70-fold less than nicotine. These findings suggest that the anxiolytic-like effect of nicotine in zebrafish was a direct effect of nicotine and not of its metabolites. Anatabine and methylanatabine were the other two alkaloids that decreased anxiety-like activity in zebrafish. Both compounds were effective at the highest concentration tested (10 mg/L). The fact that anatabine but not methylanatabine shortened the total travel time at this concentration could mean that methylanatabine is better tolerated than anatabine. The lack of significant effects observed for anabasine, nornicotine, and metanicotine may partially be attributed to their poor effectiveness in activating α4β2 nAChRs in vitro (8 ± 4% and 44 ± 17%, respectively, in preliminary data). Furthermore, the bioavailability of these compounds in the zebrafish brain was extremely poor, likely contributing to the lack of effect in the NTT. Similarly, the two nAChR reference compounds—acetylcholine and AZD1446—also were at the limit of detection in the brain. Acetylcholine, in particular, is a highly polar molecule with a charged ammonium group (Fig. 1). As a result, it would be difficult to penetrate the BBB, which may have been the reason why it was out of confidence range of the in silico model prediction. In this sense, acetylcholine served well as a negative nAChR reference compound. In comparison, AZD1446 managed to significantly reduce anxiety-like behavior in zebrafish at the lower two concentrations, despite the low predicted BBB penetration value and brain bioavailability, though the effect was rather small (~ 10%). The anxiolytic-like effect caused by AZD1446 was rather unexpected as this compound was developed as a procognitive drug and its anxiolytic effect has yet to be reported [38]. One should keep in mind, however, that the concentrations used to observe this effect were extremely high, and thus, these findings cannot be directly translated to humans.

Given that alkaloids examined in this study were all pyridine alkaloids with structurally similar subgroups, it is intriguing to find significant differences in α4β2 nAChR activation potencies and their abilities to penetrate BBB and reduce the anxiety-like behavior in zebrafish. Among the four alkaloids that showed anxiolytic-like activities, cotinine had the weakest α4β2 nAChR activity and binding in vitro and in molecular docking simulation. In fact, cotinine also only activated 59% of maximum receptor activation compared to nicotine. Perhaps, then, it was not surprising that more than 300-fold higher concentration of cotinine was needed to induce anxiolytic-like effect in zebrafish compared to nicotine. Similarly, although the EC_50_ values for α4β2 nAChR were similar among some alkaloids, the percent activation varied. For example, both nicotine and anabasine were equally potent in activating α4β2 nAChR, but anabasine activated only 7% of the maximum receptor activation compared to nicotine. This difference may have been the reason why anabasine did not induce anxiolytic-like effect in zebrafish. In addition, it is possible that other molecular targets are playing a role. For instance, many nAChR subunits, including α2, α3, α4, α7, β2, β4, can be cloned from zebrafish and the activity of key pharmacological tools like nicotine on these zebrafish nAChRs seems sufficiently similar to those on mammalian receptors [43]. A recent paper by Alijevic, et al. reported that nicotine, anatabine, and anabasine may be a weak α7 nAChR agonist, while the other alkaloids tested in this study were unable to activate α7 nAChR [2]. Interestingly, anabasine was also reported to activate α7 nAChR at approximately a third of maximum receptor activation by nicotine or anatabine [2]. Therefore, the differences of these alkaloids in regulating neurobehavioral effects may be reflected by both different levels of α4β2 nAChR activation and by nAChR subtype specificity.

In conclusion, this study is the first systematic effort to demonstrate the efficacy of various nicotinic compounds using a well-validated and accepted anxiety-like behavioral paradigm, the zebrafish NTT. We newly report that cotinine, anatabine, and methylanatabine can reduce anxiety-like activity in zebrafish, whereas other nAChR-activating alkaloids such as anabasine, nornicotine, and metanicotine cannot. Nicotine, however, was the most potent anxiolytic-like compound in the current study using zebrafish. Our findings have interestingly revealed that despite of their similar chemical structures, not all pyridine alkaloids behave the same in terms of nAChR pharmacology and BBB penetration in zebrafish, and thus, highlighting the importance of carefully investigating the natural neuroactive compounds using appropriate testing tools.

## Supplementary Information

Below is the link to the electronic supplementary material.

Supplementary file1 (PDF 34 KB)

Supplementary file2 (PDF 576 KB)

Supplementary file3 (PDF 45 KB)

Supplementary file4 (PDF 160 KB)

Supplementary file5 (PDF 140 KB)

Supplementary file6 (PDF 86 KB)

Supplementary file7 (PDF 115 KB)
