# Supplementary material for: Effects of nicotinic acetylcholine receptor-activating alkaloids on anxiety-like behavior in zebrafish
Source: J Nat Med. 2021 Jul 15;75(4):926–41. doi: 10.1007/s11418-021-01544-8 (PMC8397634; doi:10.1007/s11418-021-01544-8)
Supplement: Supplementary file 1 — Supplementary file1 (PDF 34 KB) [file 11418_2021_1544_MOESM1_ESM.pdf]

Compound	Concentration (mg/L)	Sample Size
Nicotine	0	84
	0.3	12
	1	27
	3	12
Cotinine	0	48
	30	12
	100	12
	300	24
Anatabine	0	29
	0.3	12
	1	12
	3	12
Methylanatabine	0	12
	1	12
	3	12
	10	12
Anabasine	0	12
	0.3	12
	1	12
	3	12

Compound	Concentration (mg/L)	Sample Size
Nornicotine	0	20
	3	23
	10	22
	30	22
Metanicotine	0	12
	30	12
	100	12
	300	12
Acetylcholine	0	35
	30	12
	10	18
	300	12
AZD1446	0	22
	30	24
	100	24
	300	24

Online Resource 1 Sample size per group
