# Supplementary material for: Effects of nicotinic acetylcholine receptor-activating alkaloids on anxiety-like behavior in zebrafish
Source: J Nat Med. 2021 Jul 15;75(4):926–41. doi: 10.1007/s11418-021-01544-8 (PMC8397634; doi:10.1007/s11418-021-01544-8)
Supplement: Supplementary file 2 — Supplementary file2 (PDF 576 KB) [file 11418_2021_1544_MOESM2_ESM.pdf]

3. MATERIALS AND METHODS

Some changes due to improvement of the assays may occur between the writing of this study plan and testing. Validation of these changes will include verification that they do not affect assay results. The final study report will reflect the updated experimental conditions.

3.1. Experimental Conditions

3.1.1. *In Vitro* Pharmacology: Binding Assays

Assay	Source	Ligand	Conc.	Kd	Non Specific	Incubation	Detection Method	Bibl.
**Receptors**								
**A_1*(h)*** **(antagonist radioligand)**	human recombinant (CHO cells)	[^{3}H]DPCPX	1 nM	1.7 nM	DPCPX (1 µM)	60 min RT	Scintillation counting	245
**A_1*(h)*** **(agonist radioligand)**	human recombinant (CHO cells)	[^{3}H]CCPA	1 nM	0.7 nM	CPA (10 µM)	60 min RT	Scintillation counting	198
**A_{2A}*(h)*** **(agonist radioligand)**	human recombinant (HEK-293 cells)	[^{3}H]CGS 21680	6 nM	27 nM	NECA (10 µM)	120 min RT	Scintillation counting	141
**A_3*(h)*** **(agonist radioligand)**	human recombinant (HEK-293 cells)	[^{125}I]AB-MECA	0.15 nM	0.22 nM	IB-MECA (1 µM)	120 min RT	Scintillation counting	206
**α_1 (non-selective)** **(antagonist radioligand)**	rat cerebral cortex	[^{3}H]prazosin	0.25 nM	0.09 nM	prazosin (0.5 µM)	60 min RT	Scintillation counting	88
**α_{1A}*(h)*** **(antagonist radioligand)**	human recombinant (CHO cells)	[^{3}H]prazosin	0.1 nM	0.1 nM	epinephrine (0.1 mM)	60 min RT	Scintillation counting	897
**α_{1B}*(h)*** **(antagonist radioligand)**	human recombinant (CHO cells)	[^{3}H]prazosin	0.15 nM	0.055 nM	phentolamine (10 µM)	60 min RT	Scintillation counting	701
**α_{1D}*(h)*** **(antagonist radioligand)**	human recombinant (CHO cells)	[^{3}H]prazosin	0.2 nM	0.15 nM	phentolamine (10 µM)	60 min RT	Scintillation counting	760
**α_2 (non-selective)** **(antagonist radioligand)**	rat cerebral cortex	[^{3}H]RX 821002	0.5 nM	0.38 nM	(-)epinephrine (100 µM)	60 min RT	Scintillation counting	249
**α_{2A} *(h)*** **(antagonist radioligand)**	human recombinant (CHO cells)	[^{3}H]RX 821002	1 nM	0.8 nM	(-)epinephrine (100 µM)	60 min RT	Scintillation counting	542
**α_{2B}*(h)*** **(antagonist radioligand)**	human recombinant (CHO cells)	[^{3}H]RX 821002	2.5 nM	5 nM	(-)epinephrine (100 µM)	60 min RT	Scintillation counting	56
**α_{2C} *(h)*** **(antagonist radioligand)**	human recombinant (CHO cells)	[^{3}H]RX 821002	2 nM	0.95 nM	(-)epinephrine (100 µM)	60 min RT	Scintillation counting	56
**β_1 *(h)*** **(agonist radioligand)**	human recombinant (HEK-293 cells)	[^{3}H](-)CGP 12177	0.3 nM	0.39 nM	alprenolol (50 µM)	60 min RT	Scintillation counting	548
**β_2 *(h)*** **(antagonist radioligand)**	human recombinant (CHO cells)	[^{3}H](-)CGP 12177	0.3 nM	0.15 nM	alprenolol (50 µM)	120 min RT	Scintillation counting	794
**AT_1 *(h)*** **(antagonist radioligand)**	human recombinant (HEK-293 cells)	[^{125}I][Sar^1,Ile^8]-AT-II	0.05 nM	0.05 nM	angiotensin-II (10 µM)	120 min 37°C	Scintillation counting	776
**AT_2 *(h)*** **(agonist radioligand)**	human recombinant (HEK-293 cells)	[^{125}I]CGP 42112A	0.01 nM	0.01 nM	angiotensin-II (1 µM)	4 hr 37°C	Scintillation counting	248
**APJ (apelin) *(h)*** **(agonist radioligand)**	human recombinant (CHO cells)	[^{125}I](Glp^{65},Nle^{75},Tyr^{77}) -apelin-13	0.03 nM	0.06 nM	apelin-13 (1 µM)	120 min RT	Scintillation counting	846
**B_1*(h)*** **(agonist radioligand)**	human recombinant (CHO cells)	[^{3}H]desArg^{10}-KD	0.35 nM	0.085 nM	desArg^9[Leu^8]-BK (10 µM)	60 min RT	Scintillation counting	525
**B_2 *(h)*** **(agonist radioligand)**	human recombinant (CHO cells)	[^{3}H]bradykinin	0.3 nM	0.32 nM	bradykinin (1 µM)	60 min RT	Scintillation counting	346

Assay	Source	Ligand	Conc.	Kd	Non Specific	Incubation	Detection Method	Bibl.
**CB_1*(h)*** **(agonist radioligand)**	human recombinant (CHO cells)	[^{3}H]CP 55940	0.5 nM	3.5 nM	WIN 55212-2 (10 µM)	120 min 37°C	Scintillation counting	657
**CB_2*(h)*** **(agonist radioligand)**	human recombinant (CHO cells)	[^{3}H]WIN 55212-2	0.8 nM	1.5 nM	WIN 55212-2 (5 µM)	120 min 37°C	Scintillation counting	165
**CCK_1 (CCK_A) *(h)*** **(agonist radioligand)**	human recombinant (CHO cells)	[^{125}I]CCK-8s	0.08 nM	0.24 nM	CCK-8s (1 µM)	60 min RT	Scintillation counting	562
**CCK_2 (CCK_B) *(h)*** **(agonist radioligand)**	human recombinant (CHO cells)	[^{125}I]CCK-8s	0.08 nM	0.054 nM	CCK-8s (1 µM)	60 min RT	Scintillation counting	134
**CRF_1*(h)*** **(agonist radioligand)**	human recombinant (CHO cells)	[^{125}I]sauvagine	0.075 nM	0.12 nM	sauvagine (0.5 µM)	120 min RT	Scintillation counting	557
**D_1*(h)*** **(antagonist radioligand)**	human recombinant (CHO cells)	[^{3}H]SCH 23390	0.3 nM	0.2 nM	SCH 23390 (1 µM)	60 min RT	Scintillation counting	281
**D_{2S}*(h)*** **(antagonist radioligand)**	human recombinant (HEK-293 cells)	[^{3}H]methyl-spiperone	0.3 nM	0.15 nM	(+)butaclamol (10 µM)	60 min RT	Scintillation counting	87
**D_{2S}*(h)*** **(agonist radioligand)**	human recombinant (HEK-293 cells)	[^{3}H]7-OH-DPAT	1 nM	0.68 nM	butaclamol (10 µM)	60 min RT	Scintillation counting	87
**D_{2L}*(h)*** **(antagonist radioligand)**	human recombinant (HEK-293 cells)	[^{3}H]methyl-spiperone	0.3 nM	0.1 nM	butaclamol (10 µM)	60 min RT	Scintillation counting	659
**D_3*(h)*** **(antagonist radioligand)**	human recombinant (CHO cells)	[^{3}H]methyl-spiperone	0.3 nM	0.085 nM	(+)butaclamol (10 µM)	60 min RT	Scintillation counting	145
**$D_{4.4}$*(h)*** **(antagonist radioligand)**	human recombinant (CHO cells)	[^{3}H]methyl-spiperone	0.3 nM	0.19 nM	(+)butaclamol (10 µM)	60 min RT	Scintillation counting	252
**D_5*(h)*** **(antagonist radioligand)**	human recombinant (GH4 cells)	[^{3}H]SCH 23390	0.3 nM	0.25 nM	SCH 23390 (10 µM)	60 min RT	Scintillation counting	232
**ET_A*(h)*** **(agonist radioligand)**	human recombinant (CHO cells)	[^{125}I]endothelin-1	0.03 nM	0.03 nM	endothelin-1 (100 nM)	120 min 37°C	Scintillation counting	30
**ET_B *(h)*** **(agonist radioligand)**	human recombinant (CHO cells)	[^{125}I]endothelin-1	0.03 nM	0.04 nM	endothelin-1 (0.1 µM)	120 min 37°C	Scintillation counting	541
**$GABA_{A1}$ *(h)* (α1,β2,γ2)** **(agonist radioligand)**	human recombinant (CHO cells)	[^{3}H]muscimol	15 nM	30 nM	muscimol (10 µM)	120 min RT	Scintillation counting	1096
**mGluR5 (h) (agonist radioligand)**	human recombinant (CHO cells)	[3H]Quisqualate	40nM	44nM	L-Glutamate (1mM)	120 min RT	Scintillation counting	1222
**glycine (strychnine-sensitive) (antagonist radioligand)**	rat spinal cord	[^{3}H]strychnine	2 nM	20 nM	strychnine (100 µM)	15 min 0°C	Scintillation counting	150
**CXCR2 (IL-8B) *(h)*** **(agonist radioligand)**	human recombinant (HEK-293 cells)	[^{125}I]IL-8	0.025 nM	0.022 nM	IL-8 (30 nM)	60 min RT	Scintillation counting	266
**CCR1 *(h)*** **(agonist radioligand)**	human recombinant (HEK-293 cells)	[^{125}I]MIP-1α	0.01 nM	0.02 nM	MIP-1α (100 nM)	120 min RT	Scintillation counting	172
**H_1*(h)*** **(antagonist radioligand)**	human recombinant (HEK-293 cells)	[^{3}H]pyrilamine	1 nM	1.7 nM	pyrilamine (1 µM)	60 min RT	Scintillation counting	492
**H_2*(h)*** **(antagonist radioligand)**	human recombinant (CHO cells)	[^{125}I]APT	0.075 nM	2.9 nM	tiotidine (100 µM)	120 min RT	Scintillation counting	540
**H_3*(h)*** **(agonist radioligand)**	human recombinant (CHO cells)	[^{3}H]N^{α}-Me-histamine	1 nM	0.32 nM	(R)α-Me-histamine (1 µM)	60 min RT	Scintillation counting	563
**I_2** **(antagonist radioligand)**	rat cerebral cortex	[^{3}H]idazoxan (+ 1 µM yohimbine)	2 nM	4 nM	cirazoline (10 µM)	30 min RT	Scintillation counting	27

Assay	Source	Ligand	Conc.	Kd	Non Specific	Incubation	Detection Method	Bibl.
**$CysLT_1$ (LTD_4) *(h)*<br>(agonist radioligand)**	human recombinant (CHO cells)	[^{3}H]LTD_4	0.3 nM	0.24 nM	LTD_4<br>(1 µM)	60 min<br>RT	Scintillation counting	618
**MC_1<br>(agonist radioligand)**	B16-F1 cells (endogenous)	[^{125}I]NDP-α-MSH	0.05 nM	0.05 nM	NDP-α-MSH<br>(1 µM)	90 min<br>RT	Scintillation counting	390
**MC_4*(h)*<br>(agonist radioligand)**	human recombinant (CHO cells)	[^{125}I]NDP-α-MSH	0.05 nM	0.54 nM	NDP-α-MSH<br>(1 µM)	120 min<br>37°C	Scintillation counting	211
**MC_5*(h)*<br>(agonist radioligand)**	human recombinant (CHO cells)	[^{125}I]NDP-α-MSH	0.05 nM	0.7 nM	NDP-α-MSH<br>(1 µM)	60 min<br>37°C	Scintillation counting	211
**MT_1 (ML_{1A}) *(h)*<br>(agonist radioligand)**	human recombinant (CHO cells)	[^{125}I]2-iodomelatonin	0.01 nM	0.04 nM	melatonin<br>(1 µM)	240 min<br>RT	Scintillation counting	639
**M (non-selective)<br>(antagonist radioligand)**	rat cerebral cortex	[^{3}H]QNB	0.05 nM	0.01 nM	atropine<br>(1 µM)	120 min<br>RT	Scintillation counting	195
**M_1*(h)*<br>(antagonist radioligand)**	human recombinant (CHO cells)	[^{3}H]pirenzepine	2 nM	13 nM	atropine<br>(1 µM)	60 min<br>RT	Scintillation counting	59
**M_3 *(h)*<br>(antagonist radioligand)**	human recombinant (CHO cells)	[^{3}H]4-DAMP	0.2 nM	0.5 nM	atropine<br>(1 µM)	60 min<br>RT	Scintillation counting	546
**M_4*(h)*<br>(antagonist radioligand)**	human recombinant (CHO cells)	[^{3}H]4-DAMP	0.2 nM	0.32 nM	atropine<br>(1 µM)	60 min<br>RT	Scintillation counting	59
**M_5*(h)*<br>(antagonist radioligand)**	human recombinant (CHO cells)	[^{3}H]4-DAMP	0.3 nM	0.3 nM	atropine<br>(1 µM)	60 min<br>RT	Scintillation counting	59
**NK_1*(h)*<br>(agonist radioligand)**	U373MG uppsala	[^{125}I]-Substance P LYS3	0.05 nM	0.04 nM	[Sar^9,$Met(O_2)^{11}$]-SP<br>(1 µM)	30 min<br>RT	Scintillation counting	104
**NK_2*(h)*<br>(agonist radioligand)**	human recombinant (CHO cells)	[^{125}I]NKA	0.1 nM	0.12 nM	[$Nleu^{10}$]-NKA (4-10)<br>(300 nM)	60 min<br>RT	Scintillation counting	3
**NK_3 *(h)*<br>(antagonist radioligand)**	human recombinant (CHO cells)	[^{3}H]SR 142801	0.4 nM	0.47 nM	SB 222200<br>(10 µM)	120 min<br>RT	Scintillation counting	741
**Y (non-selective)<br>(agonist radioligand)**	rat cerebral cortex	[^{125}I]peptide YY	0.05 nM	0.1 nM	NPY<br>(1 µM)	120 min<br>RT	Scintillation counting	84
**Y_1*(h)*<br>(agonist radioligand)**	SK-N-MC cells (endogenous)	[^{125}I]peptide YY	0.025 nM	0.06 nM	NPY<br>(1 µM)	120 min<br>37°C	Scintillation counting	391
**Y_2*(h)*<br>(agonist radioligand)**	KAN-TS cells	[^{125}I]peptide YY	0.015 nM	0.01 nM	NPY<br>(1 µM)	60 min<br>37°C	Scintillation counting	77
**N neuronal α4β2 *(h)*<br>(agonist radioligand)**	SH-SY5Y cells (human recombinant)	[^{3}H]cytisine	0.6 nM	0.3 nM	nicotine<br>(10 µM)	120 min<br>4°C	Scintillation counting	1084
**N neuronal α7 *(h)*<br>(antagonist radioligand)**	SH-SY5Y cells (human recombinant)	[^{125}I]α-bungarotoxin	0.05 nM	0.34 nM	α-bungarotoxin<br>(1 µM)	120 min<br>37°C	Scintillation counting	347
**N muscle-type *(h)*<br>(antagonist radioligand)**	TE671 cells (endogenous)	[^{125}I]α-bungarotoxin	0.5 nM	2 nM	α-bungarotoxin<br>(5 µM)	120 min<br>RT	Scintillation counting	524
**opioid<br>(non-selective)<br>(antagonist radioligand)**	rat cerebral cortex	[^{3}H]naloxone	1 nM	2.6 nM	naloxone<br>(1 µM)	40 min<br>RT	Scintillation counting	43
**δ (DOP) *(h)*<br>(agonist radioligand)**	recombinant humain (Chem-1)	[^{3}H]DADLE	0.5 nM	0.6 nM	naltrexone<br>(10 µM)	60 min<br>RT	Scintillation counting	501
**kappa (h) (KOP) (agonist radioligand)**	RBL recombinant	[3H]U69593	0.5 nM	0.6 nM	naloxone (10µM)	60 min<br>RT	Scintillation counting	222
**µ (MOP) *(h)*<br>(agonist radioligand)**	human recombinant (HEK-293 cells)	[^{3}H]DAMGO	0.5 nM	0.35 nM	naloxone<br>(10 µM)	120 min<br>RT	Scintillation counting	260
**NOP (ORL1) *(h)*<br>(agonist radioligand)**	recombinant humain	[^{3}H]nociceptin	0.06 nM	0.054 nM	nociceptin<br>(1 µM)	60 min<br>RT	Scintillation counting	1588
**PPARγ *(h)*<br>(agonist radioligand)**	human recombinant (*E. coli*)	[^{3}H]rosiglitazone	5 nM	5.7 nM	rosiglitazone<br>(10 µM)	120 min<br>4°C	Scintillation counting	567

Assay	Source	Ligand	Conc.	Kd	Non Specific	Incubation	Detection Method	Bibl.
**EP_2*(h)*** **(agonist radioligand)**	human recombinant (HEK-293 cells)	[^{3}H]PGE_2	3 nM	3 nM	PGE_2 (10 µM)	120 min RT	Scintillation counting	781
**IP (PGI_2) *(h)*** **(agonist radioligand)**	human recombinant (HEK-293 cells)	[^{3}H]iloprost	6 nM	8 nM	iloprost (10 µM)	60 min RT	Scintillation counting	781
**P2Y** **(agonist radioligand)**	rat cerebral cortex	[^{35}S]dATPαS	10 nM	10 nM	dATPαS (10 µM)	60 min RT	Scintillation counting	298
**$5\text{-}HT_{1A}$*(h)*** **(agonist radioligand)**	human recombinant (HEK-293 cells)	[^{3}H]8-OH-DPAT	0.5 nM	0.5 nM	8-OH-DPAT (10 µM)	60 min RT	Scintillation counting	164
**$5\text{-}HT_{1B}$** **(antagonist radioligand)**	rat cerebral cortex	[^{125}I]CYP (+ 30 µM isoproterenol)	0.1 nM	0.16 nM	serotonin (10 µM)	120 min 37°C	Scintillation counting	111
**$5\text{-}HT_{2A}$*(h)*** **(antagonist radioligand)**	human recombinant (HEK-293 cells)	[^{3}H]ketanserin	0.5 nM	0.6 nM	ketanserin (1 µM)	60 min RT	Scintillation counting	20
**$5\text{-}HT_{2A}$*(h)*** **(agonist radioligand)**	human recombinant (HEK-293 cells)	[^{125}I](±)DOI	0.1 nM	0.3 nM	(±)DOI (1 µM)	60 min RT	Scintillation counting	288
**$5\text{-}HT_{2B}$*(h)*** **(antagonist radioligand)**	human recombinant (CHO cells)	[^{3}H]mesulergine	2 nM	2.4 nM	SB206553 (10 µM)	60 min RT	Scintillation counting	678
**$5\text{-}HT_{2B}$*(h)*** **(agonist radioligand)**	human recombinant (CHO cells)	[^{125}I](±)DOI	0.2 nM	0.2 nM	(±)DOI (1 µM)	60 min RT	Scintillation counting	571
**$5\text{-}HT_{2C}$*(h)*** **(antagonist radioligand)**	human recombinant (HEK-293 cells)	[^{3}H]mesulergine	1 nM	0.5 nM	RS 102221 (10 µM)	120 min 37°C	Scintillation counting	543
**$5\text{-}HT_{2C}$*(h)*** **(agonist radioligand)**	human recombinant (HEK-293 cells)	[^{125}I](±)DOI	0.1 nM	0.9 nM	(±)DOI (10 µM)	60 min 37°C	Scintillation counting	288
**$5\text{-}HT_{4e}$*(h)*** **(antagonist radioligand)**	human recombinant (CHO cells)	[^{3}H]GR 113808	0.3 nM	0.15 nM	serotonin (100 µM)	60 min 37°C	Scintillation counting	309
**$5\text{-}HT_{5a}$ *(h)*** **(agonist radioligand)**	human recombinant (HEK-293 cells)	[^{3}H]LSD	1.5 nM	1.5 nM	serotonin (100 µM)	120 min 37°C	Scintillation counting	193
**$5\text{-}HT_6$ *(h)*** **(agonist radioligand)**	human recombinant (CHO cells)	[^{3}H]LSD	2 nM	1.8 nM	serotonin (100 µM)	120 min 37°C	Scintillation counting	161
**$5\text{-}HT_7$ *(h)*** **(agonist radioligand)**	human recombinant (CHO cells)	[^{3}H]LSD	4 nM	2.3 nM	serotonin (10 µM)	120 min RT	Scintillation counting	217
**sigma (non-selective) (h)** **(agonist radioligand)**	Jurkat cells (endogenous)	[3H]DTG	10 nM	41 nM	Haloperidol (10 µM)	120 min RT	Scintillation counting	1136
**GR *(h)*** **(agonist radioligand)**	IM-9 cells (cytosol)	[^{3}H]dexamethasone	1.5 nM	1.5 nM	triamcinolone (10 µM)	6 hr 4°C	Scintillation counting	283
**ER (non-selective) *(h)*** **(agonist radioligand)**	MCF-7 cells (cytosol)	[^{3}H]estradiol	0.4 nM	0.2 nM	17-β-estradiol (6 µM)	20 hr 4°C	Scintillation counting	1070
**Estrogen ER alpha (h)** **(agonist radioligand)**	human recombinant (sf9 cells)	[3H] Estradiol	0.5 nM	0.20 nM	Diethylstilbestrol (1µM)	120 min RT	Scintillation counting	1280
**PR *(h)*** **(agonist radioligand)**	T47D cells (cytosol)	[^{3}H]progesterone	0.5 nM	2 nM	promegestone (1 µM)	20 hr 4°C	Scintillation counting	930
**AR *(h)*** **(agonist radioligand)**	LNCaP cells (cytosol)	[^{3}H]methyltrienolone	1 nM	0.8 nM	testostérone (1 µM)	24 hr 4°C	Scintillation counting	498
**TRH_1*(h)*** **(agonist radioligand)**	human recombinant (CHO cells)	[^{3}H]Me-TRH	2 nM	3.9 nM	TRH (10 µM)	120 min 4°C	Scintillation counting	709
**V_{1a}*(h)*** **(agonist radioligand)**	human recombinant (CHO cells)	[^{3}H]AVP	0.3 nM	0.5 nM	AVP (1 µM)	60 min RT	Scintillation counting	343
**V_2*(h)*** **(agonist radioligand)**	human recombinant (CHO cells)	[^{3}H]AVP	0.3 nM	0.76 nM	AVP (1 µM)	120 min RT	Scintillation counting	343

Assay	Source	Ligand	Conc.	Kd	Non Specific	Incubation	Detection Method	Bibl.
**Ion channels**								
**BZD (central) (agonist radioligand)**	rat cerebral cortex	[^{3}H]flunitrazepam	0.4 nM	2.1 nM	diazepam (3 µM)	60 min 4°C	Scintillation counting	227
**AMPA (agonist radioligand)**	rat cerebral cortex	[^{3}H]AMPA	8 nM	82 nM	L-glutamate (1 mM)	60 min 4°C	Scintillation counting	166
**kainate (agonist radioligand)**	rat cerebral cortex	[^{3}H]kainic acid	5 nM	19 nM	L-glutamate (1 mM)	60 min 4°C	Scintillation counting	160
**glycine (strychnine-insensitive) (antagonist radioligand)**	rat cerebral cortex	[^{3}H]MDL 105,519	0.5 nM	5 nM	glycine (1 mM)	45 min 0°C	Scintillation counting	219
**P2X (agonist radioligand)**	rat urinary bladder	[^{3}H]α,β-MeATP	3 nM	2.6 nM	α,β-MeATP (10 µM)	120 min 4°C	Scintillation counting	17
**5-HT$_3$*(h)* (antagonist radioligand)**	human recombinant (CHO cells)	[^{3}H]BRL 43694	0.5 nM	1.15 nM	MDL 72222 (10 µM)	120 min RT	Scintillation counting	109
**Ca^{2+} channel (L, dihydropyridine site) (antagonist radioligand)**	rat cerebral cortex	[^{3}H]nitrendipine	0.1 nM	0.18 nM	nitrendipine (1 µM)	90 min RT	Scintillation counting	996
**Ca^{2+} channel (L, diltiazem site) (benzothiazepines) (antagonist radioligand)**	rat cerebral cortex	[^{3}H]diltiazem	15 nM	52 nM	diltiazem (10 µM)	120 min RT	Scintillation counting	212
**Ca^{2+} channel (L, verapamil site) (phenylalkylamine) (antagonist radioligand)**	rat cerebral cortex	[^{3}H]D888	3 nM	3 nM	D 600 (10 µM)	120 min RT	Scintillation counting	194
**Ca^{2+} channel (N) (antagonist radioligand)**	rat cerebral cortex	[^{125}I]ω-conotoxin GVIA	0.001 nM	0.0007 nM	ω-conotoxin GVIA (10 nM)	30 min RT	Scintillation counting	259
**K_{ATP} channel (antagonist radioligand)**	rat cerebral cortex	[^{3}H]glibenclamide	0.1 nM	0.05 nM	glibenclamide (1 µM)	60 min RT	Scintillation counting	6
**Potassium Channel hERG (human)- [3H] Dofetilide**	human recombinant (HEK-293 cells)	[3H]Dofetilide	3 nM	6.6 nM	Terfenadine (25 µM)	60 min RT	Scintillation counting	1398
**K_V channel (antagonist radioligand)**	rat cerebral cortex	[^{125}I]α-dendrotoxin	0.01 nM	0.04 nM	α-dendrotoxin (50 nM)	60 min RT	Scintillation counting	225
**SK_{Ca} channel (antagonist radioligand)**	rat cerebral cortex	[^{125}I]apamin	0.007 nM	0.007 nM	apamin (100 nM)	60 min 4°C	Scintillation counting	112
**Na^+ channel (site 2) (antagonist radioligand)**	rat cerebral cortex	[^{3}H]batrachotoxin	10 nM	91 nM	veratridine (300 µM)	60 min 37°C	Scintillation counting	28
**Transporters**								
**adenosine transporter (antagonist radioligand)**	guinea-pig cerebral cortex	[^{3}H]NBTI	0.15 nM	0.08 nM	NBTI (5 µM)	30 min RT	Scintillation counting	254
**norepinephrine transporter *(h)* (antagonist radioligand)**	human recombinant (CHO cells)	[^{3}H]nisoxetine	1 nM	2.9 nM	desipramine (1 µM)	120 min 4°C	Scintillation counting	180
**dopamine transporter *(h)* (antagonist radioligand)**	human recombinant (CHO cells)	[^{3}H]BTCP	4 nM	4.5 nM	BTCP (10 µM)	120 min 4°C	Scintillation counting	190
**GABA transporter (antagonist radioligand)**	rat cerebral cortex	[^{3}H]GABA (+ 10 µM isoguvacine) (+ 10 µM baclofen)	10 nM	4600 nM	GABA (1 mM)	30 min RT	Scintillation counting	214
**choline transporter (CHT1) *(h)* (antagonist radioligand)**	human recombinant (CHO cells)	[^{3}H]hemicholinium-3	3 nM	3.9 nM	hemicholinium-3 (10 µM)	60 min RT	Scintillation counting	648
**5-HT transporter *(h)* (antagonist radioligand)**	human recombinant (CHO cells)	[^{3}H]imipramine	2 nM	1.7 nM	imipramine (10 µM)	60 min RT	Scintillation counting	566

3.1.2. *In Vitro* Pharmacology: Cellular and Nuclear Receptor Functional Assays

Assay	Source	Stimulus	Incubation	Measured Component	Detection Method	Bibl.
**Receptors**						
**CT (Calcitonin) *(h)* (agonist effect)**	T47D cells (endogenous)	none (1 µM human calcitonin for control)	10 min RT	cAMP	HTRF	803
**CT (Calcitonin) *(h)* (antagonist effect)**	T47D cells (endogenous)	human calcitonin (30 nM)	10 min RT	cAMP	HTRF	803
**GPR109A *(h)* (agonist effect)**	**human recombinant (RBL cells)**	none (12 µM Nicotinic Acid Free Acid for control)	RT	intracellular [Ca^{2+}]	Fluorimetry	1528
**GPR109A *(h)* (antagonist effect)**	**human recombinant (RBL cells)**	Nicotinic Acid Free Acid (300 nM)	RT	intracellular [Ca^{2+}]	Fluorimetry	1528
**P2Y11 *(h)* (agonist effect)**	human recombinant (1321N1 cells)	none (1 mM of ATP for control)	RT	intracellular [Ca^{2+}]	Fluorimetry	1531
**P2Y11 *(h)* (antagonist effect)**	human recombinant (1321N1 cells)	ATP (60 µM)	RT	intracellular [Ca^{2+}]	Fluorimetry	1531

3.1.3. *In Vitro* Pharmacology: Enzyme and Uptake Assays

Assay	Source	Substrate/Stimulus/Tracer	Incubation	Measured Component	Detection Method	Bibl.
**Kinases**						
**Abl kinase *(h)***	human recombinant (insect cells)	ATP + Ulight-TK peptide (100 nM)	30 min RT	phospho-Ulight-TK peptide	LANCE	556
**ERK_1*(h)***	human recombinant (*E. coli*)	ATP + Ulight-CFFKNIVTPRTPPPSQGK-amide (50 nM)	30 min RT	phospho-Ulight-CFFKNIVTPRTPPPSQGK-amide	LANCE	713
**ERK_2*(h)* ($P42^{mapk}$)**	human recombinant (*E. coli*)	ATP + Ulight-CFFKNIVTPRTPPPSQGK-amide (100 nM)	15 min RT	phospho-Ulight-CFFKNIVTPRTPPPSQGK-amide	LANCE	671
**Fyn kinase *(h)***	human recombinant (insect cells)	ATP + biotinyl-βAβAβAYQAEENTYDEYEN (2 µM)	60 min RT	phospho-biotinyl-βAβAβAYQAEENTYDEYEN	HTRF	626
**HER2/ErbB2 kinase *(h)***	human recombinant (insect cells)	ATP + biotinyl-βAβAβAAEEEEYFELVAKKK (600 nM)	10 min RT	phospho-biotinyl-βAβAβAAEEEEYFELVAKKK	HTRF	681
**IRK *(h)* (InsR)**	human recombinant	ATP + Ulight-Poly GAT[EAY(1:1:1)]n (50 nM)	10 min RT	phospho-Ulight-Poly GAT[EAY(1:1:1)]n	LANCE	467
**JNK1 *(h)***	human recombinant (*E. coli*)	ATP + Ulight-CFFKNIVTPRTPPPSQGK-amide (100 nM)	60 min RT	phospho-Ulight-CFFKNIVTPRTPPPSQGK-amide	LANCE	611
**JNK3 *(h)***	human recombinant (*E. coli*)	ATP + Ulight-CFFKNIVTPRTPPPSQGK-amide (100 nM)	30 min RT	phospho-Ulight-CFFKNIVTPRTPPPSQGK-amide	LANCE	680
**Lck kinase *(h)***	human recombinant (insect cells)	ATP + Ulight-Poly GAT[EAY(1:1:1)]n (25 nM)	10 min RT	phospho-Ulight-Poly GAT[EAY(1:1:1)]n	LANCE	556
**Lyn A kinase *(h)***	human recombinant (insect cells)	ATP + biotinyl-βAβAβAKVEKIGEGTYGVVYK (400 nM)	120 min RT	phospho-biotinyl-βAβAβAKVEKIGEGTYGVVYK	HTRF	41
**p38α kinase *(h)***	human recombinant (*E. coli*)	ATP + Ulight-CFFKNIVTPRTPPPSQGK-amide (100 nM)	30 min RT	phospho-Ulight-CFFKNIVTPRTPPPSQGK-amide	LANCE	620
**PKCα *(h)***	human recombinant (insect cells)	ATP + biotinyl-βAβAβAKIQASFRGHMARKK (60 nM)	10 min RT	phospho-biotinyl-βAβAβAKIQASFRGHMARKK	HTRF	628
**Src kinase *(h)***	human recombinant (insect cells)	ATP + Ulight-Poly GAT[EAY(1:1:1)]n (5 nM)	10 min RT	phospho-Ulight-Poly GAT[EAY(1:1:1)]n	LANCE	41
**Epigenetic enzymes and DNA-related enzymes**						
**Catechol O-methyltransferase (h)**	human recombinant (E. coli)	Pyrocatechol (15 µM)/ SAM (10 µM)	15 min, 37°C	SAH	MS	1491
**CENP-E *(h)***	human recombinant (*E. coli*)	ATP (100 µM)	5 min RT	inorganic phosphate	Photometry	853
**Eg5 *(h)***	human recombinant	ATP (50 µM)	10 min RT	inorganic phosphate	Photometry	853
**HDAC3 *(h)***	human recombinant	fluorogenic HDAC substrate (50 µM)	10 min RT	fluoro-lysine	Fluorimetry	896
**HDAC4 *(h)***	human recombinant	fluorogenic HDAC substrate class 2a (20 µM)	30 min RT	fluoro-lysine	Fluorimetry	896
**HDAC6 *(h)***	human recombinant	fluorogenic HDAC substrate (25 µM)	30 min RT	fluoro-lysine	Fluorimetry	896
**HDAC11 *(h)***	human recombinant	fluorogenic HDAC substrate class 2a (50 µM)	30 min 37°C	fluoro-lysine	Fluorimetry	896
**sirtuin 1 *(h)* (activator effect)**	human recombinant (*E. coli*)	fluorogenic HDAC substrate (200 µM)	20 min RT	fluoro-lysine	Fluorimetry	976
**sirtuin 2 *(h)* (inhibitor effect)**	human recombinant (*E. coli*)	fluoro-lysine sirtuin 2 deacetylase substrate (150 µM)	60 min RT	fluoro-lysine	Fluorimetry	976

Assay	Source	Substrate/Stimulus/Tracer	Incubation	Measured Component	Detection Method	Bibl.
**Other enzymes**						
**Cholinesterase Butyryl CHLE (h)**	human serum	S butyrylthiocholine (0.56 mM)	120 min RT	5 thio 2 nitro benzoic acid	Photometry	1218
**COX1(h)**	human recombinant	Arachidonic acid (3µM) + ADHP (25 µM)	3 min RT	Resorufin (oxydized ADHP)	Fluorimetry	1480
**COX2(h)**	human recombinant (Sf9 cells)	arachidonic acid (1.2 µM)+ ADHP (25 µM)	5 min RT	Resorufin (oxydized ADHP)	Fluorimetry	1480
**5-lipoxygenase *(h)***	human recombinant (Sf9 cells) (cytosol)	arachidonic acid (25 µM)	20 min RT	rhodamine 123	Fluorimetry	1068
**PDE1B *(h)***	human recombinant (Sf9 cells)	[3H]cGMP + cGMP (1.5 µM)	20 min RT	[3H]5'GMP	Scintillation counting	1399
**PDE2A1 *(h)***	human recombinant (Sf9 cells)	[3H]cAMP + cAMP (2µM)	20 min RT	[3H]5'AMP	Scintillation counting	1399
**PDE3A *(h)***	human recombinant (Sf9 cells)	[3H]cAMP + cAMP (0.5µM)	20 min RT	[3H]5'AMP	Scintillation counting	1399
**PDE4A1A *(h)***	human recombinant (Sf9 cells)	[3H]cAMP + cAMP (0.5µM)	20 min RT	[3H]5'AMP	Scintillation counting	1399
**PDE4B1 *(h)***	human recombinant (Sf9 cells)	[3H]cAMP + cAMP (0.5µM)	20 min RT	[3H]5'AMP	Scintillation counting	1399
**PDE4D2 *(h)***	human recombinant (Sf9 cells)	[3H]cAMP + cAMP (0.5µM)	20 min RT	[3H]5'AMP	Scintillation counting	1399
**PDE5 *(h)* (non-selective)**	human platelets	[^{3}H]cGMP + cGMP (1 µM)	60 min RT	[^{3}H]5'GMP	Scintillation counting	263
**ACE *(h)***	human recombinant	Abz-FRK(Dnp)-P-OH (15 µM)	30 min 37°C	Abz-Phe-Arg	Fluorimetry	1128
**elastase *(h)***	human neutrophil	MeOSAAPV-pNa (0.4 mM)	30 min 37°C	pNa	Photometry	2
**cathepsin G *(h)***	human leukocytes	N-SAAPF-pNa (1 mM)	15 min 37°C	pNa	Photometry	169
**MMP-1 *(h)***	human recombinant (*E. coli*)	DNP-Pro-Cha-Gly-Cys(Me)-His-Ala-Lys(n-Me-Abz)-NH_2 (10 µM)	20 min 37°C	Cys(Me)-His-Ala-Lys(n-Me-Abz)-NH_2	Fluorimetry	342
**MMP-9 *(h)***	human recombinant	NFF-2 (10 µM)	45 min 37°C	Mca-Arg-Pro-Lys-Pro-Tyr-Ala	Fluorimetry	297
**adenylyl cyclase (activator effect)**	CHO cells	none (100 µM forskolin for control)	10 min RT	cAMP	HTRF	1109
**guanylyl cyclase *(h)* (activator effect)**	human recombinant	GTP (10 µM) (100 µM SNP for control)	10 min RT	cGMP	HTRF	1076
**phosphatase 1B *(h)* (PTP1B)**	human recombinant (*E. coli*)	DIFMUP (10 µM)	30 min RT	DIFMU	Fluorimetry	928
**acetylcholinesterase *(h)***	human recombinant (HEK-293 cells)	Acetylthiocholine (400 µM)	30 min RT	5 thio 2 nitrobenzoic acid	Photometry	63
**MAO-A *(h)***	human placenta	kynuramine (0.15 mM)	20 min RT	4-OHquinoline	Photometry	265
**MAO-B *(h)* recombinant enzyme**	human recombinant	D-Luciferin derivative (4 µM)	60 min 37°C	methyl ester luciferin	Luminescence	1134
**tyrosine hydroxylase**	rat striatum	[^{3}H]tyrosine (10 µM)	20 min 37°C	[^{3}H]H_2O	Scintillation counting	168
**ATPase (Na^+/K^+)**	porcine cerebral cortex	ATP (2 mM)	60 min 37°C	Pi	Photometry	71

Assay	Source	Substrate/Stimulus/Tracer	Incubation	Measured Component	Detection Method	Bibl.

4. BIBLIOGRAPHY

2. Adeyemi, E.O. et al. (1990), *J. Pharm. Pharmacol.,* 42 *: 487-490.*
3. Aharony, D. et al. (1993), *Mol. Pharmacol.,* 44 *: 356-363.*
6. Angel, I. and Bidet, S. (1991), *Fundam. Clin. Pharmacol.,* 5 *: 107-115.*
17. Bo, X. and Burnstock, G. (1990), *Brit. J. Pharmacol.,* 101 *: 291-296.*
20. Bonhaus, D.W. et al. (1995), *Brit. J. Pharmacol.,* 115 *: 622-628.*
27. Brown, C.M. et al. (1990), *Brit. J. Pharmacol.,* 99 *: 803-809.*
28. Brown, G.B. (1986), *J. Neurosci.,* 6 *: 2064-2070.*
30. Buchan, K.W. et al. (1994), *Brit. J. Pharmacol.,* 112 *: 1251-1257.*
41. Cheng , H.C. et al. (1992), *J. Biol. Chem.,* 267 *: 9248-9256.*
43. Childers, S.R. et al. (1979), *Eur. J. Pharmacol.,* 55 *: 11-18.*
56. Devedjian, J.C. et al. (1994), *Eur. J. Pharmacol.,* 252 *: 43-49.*
59. Dorje, F. et al. (1991), *J. Pharmacol. Exp. Ther.,* 256 *: 727-733.*
63. Ellman, G.L. et al. (1961), *Biochem. Pharmacol.,* 7 *: 88-95.*
71. Fiske, C.M. and Subbarow, Y. (1925), *J. Biol. Chem.,* 66 *: 375-400.*
77. Fuhlendorff, J. et al. (1990), *Proc. Natl. Acad. Sci. U.S.A.,* 87 *: 182-186.*
84. Goldstein, M. et al. (1986), *Progr. Brain Res.,* 68 *: 331-335.*
87. Grandy, D.K. et al. (1989), *Proc. Natl. Acad. Sci. U.S.A.,* 86 *: 9762-9766.*
88. Greengrass, P. and Bremner, R. (1979), *Eur. J. Pharmacol.,* 55 *: 323-326.*
104. Heuillet, E. et al. (1993), *J. Neurochem.,* 60 *: 868-876.*
109. Hope, A.G. et al. (1996), *Brit. J. Pharmacol.,* 118 *: 1237-1245.*
111. Hoyer, D. et al. (1985), *Eur. J. Pharmacol.,* 118 *: 1-12.*
112. Hugues, M. et al. (1982), *J. Biol. Chem.,* 257 *: 2762-2769.*
134. Lee, Y.M. et al. (1993), *J. Biol. Chem.,* 268 *: 8164-8169.*
141. Luthin, D.R. et al. (1995), *Mol. Pharmacol.,* 47 *: 307-313.*
145. Mackenzie, R.G. et al. (1994), *Eur. J. Pharmacol.,* 266 *: 79-85.*
150. Marvizon, J.C. et al. (1986), *Mol. Pharmacol.,* 30 *: 590-597.*
160. Monaghan, D.T. and Cotman, C.W. (1982), *Brain Res.,* 252 *: 91-100.*
161. Monsma, F.J. et al. (1993), *Mol. Pharmacol.,* 43 *: 320-327.*
164. Mulheron, J.G. et al. (1994), *J. Biol. Chem.,* 269 *: 12954-12962.*
165. Munro, S. et al. (1993), *Nature,* 365 *: 61-65.*
166. Murphy, D.E. et al. (1987), *Neurochem. Res.,* 12 *: 775-781.*
168. Nagatsu, T. et al. (1964), *Anal. Biochem.,* 9 *: 122-126.*
169. Nakajima, K. and Powers, J. C. (1979), *J. Biol. Chem.,* 254 *: 4027-4032.*
172. Neote, K. et al. (1993), *Cell,* 72 *: 415-425.*
180. Pacholczyk, T. et al. (1991), *Nature,* 350 *: 350-354.*
190. Pristupa, Z.B. et al. (1994), *Mol. Pharmacol.,* 45 *: 125-135.*
193. Rees, S. et al. (1994), *FEBS Lett.,* 355 *: 242-246.*
194. Reynolds, I.J. et al. (1986), *J. Pharmacol. Exp. Ther.,* 237 *: 731-738.*
195. Richards, M.H. (1990), *Brit. J. Pharmacol.,* 99 *: 753-761.*
198. Rivkees, S.A. et al. (1995), *J. Biol. Chem.,* 270 *: 20485-20490.*
206. Salvatore, C.A. et al. (1993), *Proc. Natl. Acad. Sci. U.S.A.,* 90 *: 10365-10369.*
211. Schioth, H.B. et al. (1997), *Neuropeptides,* 31 *: 565-571.*
212. Schoemaker, H. and Langer, S.Z. (1985), *Eur. J. Pharmacol.,* 111 *: 273-277.*
214. Shank, R.P. et al. (1990), *J. Neurochem.,* 54 *: 2007-2015.*
217. Shen, Y. et al. (1993), *J. Biol. Chem.,* 268 *: 18200-18204.*
219. Baron, B.M. et al. (1996), *J. Pharmacol. Exp. Ther.,* 279 *: 62-68.*
222. Simonin, F. et al. (1995), *Proc. Natl. Acad. Sci. U.S.A.,* 92 *: 7006-7010.*
225. Sorensen, R.G. and Blaustein, M.P. (1989), *Mol. Pharmacol.,* 36 *: 689-698.*
227. Speth, R.C. et al. (1979), *Life Sci.,* 24 *: 351-358.*
232. Sunahara, R.K. et al. (1991), *Nature,* 350 *: 614-619.*
245. Townsend-Nicholson, A. and Schofield, P.R. (1994), *J. Biol. Chem.,* 269 *: 2373-2376.*
248. Tsuzuki, S. et al. (1994), *Biochem. Biophys. Res. Commun.,* 200 *: 1449-1454.*
249. Uhlen, S. and Wikberg, J.E. (1991), *Pharmacol. Toxicol.,* 69 *: 341-350.*
252. Van Tol, H.H.M. et al. (1992), *Nature,* 358 *: 149-152.*
254. Verma, A. and Marangos, P.J. (1985), *Life Sci.,* 36 *: 286-290.*
259. Wagner, J.A. et al. (1988), *J. Neurosci.,* 8 *: 3354-3359.*
260. Wang, J.B. et al. (1994), *FEBS Lett.,* 338 *: 217-222.*
263. Weishaar, R.E. et al. (1986), *Biochem. Pharmacol.,* 35 *: 787-800.*
265. Weyler, W. and Salach, J.I. (1985), *J. Biol. Chem.,* 260 *: 13199-13207.*
266. White, J.R. et al. (1998), *J. Biol. Chem.,* 273 *: 10095-10098.*
281. Zhou, Q.Y. et al. (1990), *Nature,* 347 *: 76-80.*
283. Clark, A.F. et al. (1996), *Invest. Ophtalmol. Vis. Sci.,* 37 *: 805-813.*
288. Bryant, H.U. et al. (1996), *Life Sci.,* 15 *: 1259-1268.*
297. Nagase, N. et al. (1994), *J. Biol. Chem.,* 269 *: 20952-20957.*
298. Simon, J. et al. (1995), *Pharmacol. Toxicol.,* 76 *: 302-307.*
309. Mialet, J. et al. (2000), *Brit. J. Pharmacol.,* 129 *: 771-781.*
342. Bickett, D.A. et al. (1993), *Anal. Biochem.,* 212 *: 58-64.*
343. Tahara, A. et al. (1998), *Brit. J. Pharmacol.,* 125 *: 1463-1470.*
346. Pruneau, D. et al. (1998), *Brit. J. Pharmacol.,* 125 *: 365-372.*
347. Sharples, C.G.V. et al. (2000), *J. Neurosci.,* 20 *: 2783-2791.*
390. Siegrist, W. et al. (1988), *J. Recep. Res.,* 8 *: 323-343.*
391. Wieland, H. A. et al. (1995), *J. Pharmacol. Exp. Ther.,* 275 *: 143-149.*
467. Al-Hasani, H. et al. (1994), *FEBS Lett.,* 349 *: 17-22.*
492. Smit, M.J. et al. (1996), *Brit. J. Pharmacol.,* 117 *: 1071-1080.*
498. Zava, D.T. et al. (1979), *Endocrinology,* 104 *: 1007-1012.*
501. Simonin, F. et al. (1994), *Mol. Pharmacol.,* 46 *: 1015-1021.*
524. Lukas, R.J. (1986), *J. Neurochem.,* 46 *: 1936-1941.*
525. Jones, C. et al. (1999), *Eur. J. Pharmacol.,* 374 *: 423-433.*
540. Leurs, R. et al. (1994), *Brit. J. Pharmacol.,* 112 *: 847-854.*
541. Fuchs, S. et al. (2001), *Mol. Med.,* 7 *: 115-124.*
542. Langin, D. et al. (1989), *Eur. J. Pharmacol.,* 167 *: 95-104.*
543. Stam, N.J. et al. (1994), *Eur. J. Pharmacol.,* 269 *: 339-348.*
546. Peralta, E. G. et al. (1987), *Embo. J.,* 6 *: 3923-3929.*
548. Levin, M.C. et al. (2002), *J. Biol.Chem.,* 277 *: 30429-30435.*
556. Park, Y.M. et al. (1999), *Anal. Biochem.,* 269 *: 94-104.*
557. Palchaudhuri, M.R. et al. (1998), *Eur. J. Biochem.,* 258 *: 78-84.*
562. Bignon, E. et al. (1999), *J. Pharmacol. Exp. Ther.* 289 *: 742-751.*
563. Lovenberg, T.W. et al. (1999), *Mol. Pharmacol.,* 55 *: 1101-1107.*
566. Tatsumi, M. et al. (1999), *Eur. J. Pharmacol.,* 368 *: 277-283.*
567. Ferry, G. et al. (2001), *Eur. J. Pharmacol.,* 417 *: 77-89.*
571. Choi, D.S. et al. (1994), *FEBS Lett.,* 352 *: 393-399.*
611. Bennett, B.L. et al. (2001), *Proc. Natl. Acad. Sci. U.S.A.,* 98 *: 13681-13686.*
618. Martin, V. et al. (2001), *Biochem. Pharmacol.,* 62 *: 1193-1200.*
620. Frantz, B. et al. (1998), *Biochemistry,* 37 *: 13846-13853.*
626. Dente, L. et al. (1997), *J. Mol. Biol.,* 269 *: 694-703.*
628. Chen, S.J. et al. (1993), *Biochemistry,* 32 *: 1032-1039.*
639. Witt-Enderby, P.A. and Dubocovich, M.L. (1996), *Mol. Pharmacol.,* 50 *: 166-174.*
648. Apparsundaram, S. et al. (2000), *Biochem. Biophys. Res. Commun.,* 276 *: 862-867.*
657. Rinaldi-Carmona, M. et al. (1996), *J. Pharmacol. Exp. Ther.,* 278 *: 871-878.*
659. Hall, D.A. and Strange, P.G. (1997), *Brit. J. Pharmacol.,* 121 *: 731-736.*
671. Bardwell, A.J. et al. (2003), *Biochem. J.,* 370 *: 1077-1085.*
678. Kursar, J.D. et al. (1994), *Mol. Pharmacol.,* 46 *: 227-234.*
680. Lisnock, J.M. et al. (2000), *Biochemistry,* 39 *: 3141-3148.*
681. Qian, X. et al. (1992), *Proc. Natl. Acad. Sci. USA,* 89 *: 1330-1334.*
701. Ford, A.P.D.W. et al. (1997), *Brit. J. Pharmacol.,* 121 *: 1127-1135.*
709. Hinuma, S. et al. (1994), *Biochem. Biophys. Acta.,* 1219 *: 251-259.*
713. Yung, Y. et al. (2001), *J. Biol. Chem.,* 276 *: 35280-35289.*
741. Sarau, H.M. et al. (1997), *J. Pharmacol. Exp. Ther.,* 281 *: 1303-1311.*
760. Kenny, B.A. et al. (1995), *Brit. J. Pharmacol.,* 115 *: 981-986.*
776. Le, M.T. et al. (2005), *Eur. J. Pharmacol.,* 513 *: 35-45.*
781. Abramovitz, M. et al. (2000), *Biochem. Biophys. Acta.,* 1483 *: 285-293.*
794. Joseph, S.S. et al. (2004), *Naun.-Sch. Arch. Pharm.,* 369 *: 525-532.*
803. Zimmermann, U. et al. (1997), *J. Endocrinol.,* 155 *: 423-431.*
846. Katugampola, S.D. et al. (2001), *Brit. J. Pharmacol.,* 132 *: 1255-1260.*
853. Goldstein, L.S.B (1993), *An. Rev. Genetics,* 27 *: 319-351.*
896. Strahl, B.D. and Allis, C.D. (2000), *Nature,* 403 *: 41-45.*
897. Schwinn, D.A. et al. (1990), *J. Biol. Chem.,* 265 *: 8183-8189.*
928. Welte, S. et al. (2005), *Anal. Biochem.,* 338 *: 32-38.*
930. Sarup, J.C. et al. (1988), *J. Biol. Chem.,* 263 *: 5624-5633.*
976. Michan, S. and Sinclair, D. (2007), *Biochem. J.,* 404 *: 1-13.*
996. Gould, R.J. et al. (1982), *Proc. Natl. Acad. Sci. U S A.,* 79 *: 3656-3660.*
1068. Pufahl, R.A. et al. (2007), *Anal. Biochem.,* 364 *: 204-212.*
1070. Kurata, Y. et al. (2005), *J. Pharmacol. Exp. Ther.,* 313 *: 916-920.*

1076. Lee, Y.C. et al. (2000), *Proc. Natl. Acad. Sci. USA,* 20 *: 10763-10768.*
1084. Gopalakrishnan, M. et al. (1996), *J. Pharmacol. Exp. Ther.,* 276 *: 289-297.*
1096. Wang, X.K. (2001), *Acta. Pharmacol. Sin.,* 22 *: 521-523.*
1109. Johnson, R.A. et al. (1997), *J. Biol. Chem.,* 272 *: 8962-8966.*
1128. Fernandes, T. et al. (2010), *Braz J Med Biol Res.,* 43 *: 837-842*
1134. Tsugeno, Y. et al. (1995), *J.Biochem., 1995;* 118 *(5) 974-80*
1136. GANAPATHY ME. et al. (1999), *JPET,* 289 *: 251-260*
1218. Alcala Mdel M. et al. (2003), *Neuropharmacology. 44(6): 749-755*
1222. Mutel, V. et al. (2000), *J.Neurochem., 75: 2590-2601*
1280. Obourn JD, Koszewski NJ and Notides AC (1993), Obourn JD et al. Biochemistry 32(24):6229, 1993.
1398. Huang XP1, Mangano T, Hufeisen S, Setola V, Roth BL., *Assay Drug Dev Technol. 2010 Dec;8(6):727-42*
1399. Maurice D.H. et al. (2014), *Nat Rev Drug Discov.,* 13: *290-314*
1480. Pattaraporn Vanachayangkul and William H.Tolleson (2012), *Hindawi Publishing Corporation, Enzyme Research, Volume 2012, Article ID 416062, 7*
1491. akub Krijt, Alena Dutá, and Viktor Kozich (2009), *J Chromatogr B Analyt Technol Biomed Life Sci.; 877(22-3): 2061-2066*
1528. Altschul R et al. (1955), *Arch. Biochem.* 54 *: 558-559*
1531. Abbracchio MP et al. (2006), *Pharmacol. Rev.* 58 *: 281-341*
1588. 1. Mogil J.S. and Pasternak G.W. (2001), Pharmacol. Rev. 53: 381-415.
