# Supplementary material for: Effects of nicotinic acetylcholine receptor-activating alkaloids on anxiety-like behavior in zebrafish
Source: J Nat Med. 2021 Jul 15;75(4):926–41. doi: 10.1007/s11418-021-01544-8 (PMC8397634; doi:10.1007/s11418-021-01544-8)
Supplement: Supplementary file 3 — Supplementary file3 (PDF 45 KB) [file 11418_2021_1544_MOESM3_ESM.pdf]

Compound	α4β2 nAChR EC_{50} (μM)	% activation (normalized to Nicotine)
Nicotine	0.8 ± 0.1	100 %
Cotinine	85.3 ± 13.4	59 %
Anatabine	5.4 ± 1.1	80 %
Methylanatabine	6.2 ± 2.1	26 %
Anabasine	0.9 ± 0.2	7 %
Nornicotine	4.9 ± 0.7	44 %
Metanicotine	19.5 ± 6.4	100 %
Acetylcholine	1.0 ± 1.0	100 %
AZD1446	7.9 ± 3.6	100 %

Online Resource 3 Summary of α4β2 nAChR EC_{50} and percent activation
