# Supplementary material for: Effects of nicotinic acetylcholine receptor-activating alkaloids on anxiety-like behavior in zebrafish
Source: J Nat Med. 2021 Jul 15;75(4):926–41. doi: 10.1007/s11418-021-01544-8 (PMC8397634; doi:10.1007/s11418-021-01544-8)
Supplement: Supplementary file 5 — Supplementary file5 (PDF 140 KB) [file 11418_2021_1544_MOESM5_ESM.pdf]

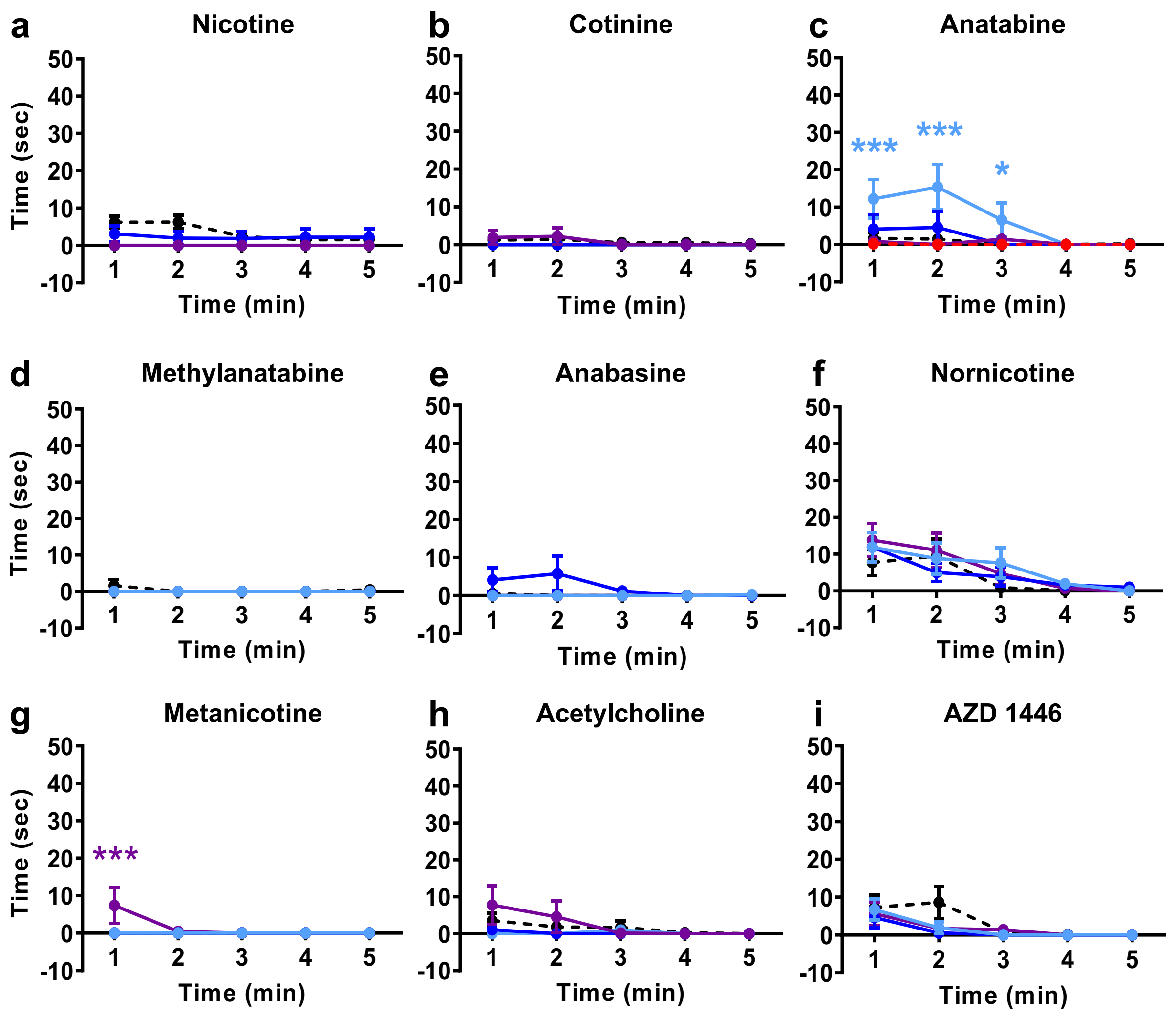

Online Resource 5 Effects of compounds on freezing response

Total freezing time during the NTT is presented for (a) nicotine (0.3, 1, and 3 mg/L), (b) cotinine (30, 100, and 300 mg/L), (c) anatabine (0.3, 1, 3, and 10 mg/L), (d) methylanatabine (1, 3, and 10 mg/L), (e) anabasine (0.3, 1, and 3 mg/L), (f) nornicotine (3, 10, and 30 mg/L), (g) metanicotine (30, 100, and 300 mg/L), (h) acetylcholine (30, 100, and 300 mg/L), and (i) AZD1446 (30, 100, and 300 mg/L). Black dashed lines = control; light blue = lowest concentration; blue = middle concentration; purple = highest concentration. For anatabine only, red is the highest concentration. *$p<0.05$; ***$p<0.001$. Data are expressed as mean ± SEM.
