# Supplementary material for: Effects of nicotinic acetylcholine receptor-activating alkaloids on anxiety-like behavior in zebrafish
Source: J Nat Med. 2021 Jul 15;75(4):926–41. doi: 10.1007/s11418-021-01544-8 (PMC8397634; doi:10.1007/s11418-021-01544-8)
Supplement: Supplementary file 6 — Supplementary file6 (PDF 86 KB) [file 11418_2021_1544_MOESM6_ESM.pdf]

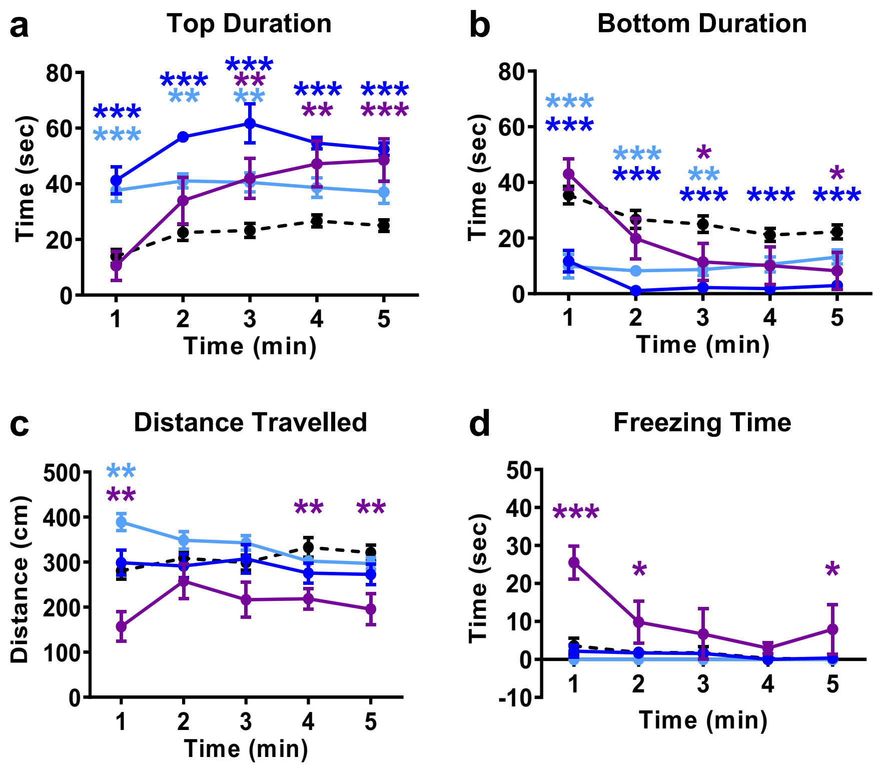

Online Resource 6 Effect of buspirone on zebrafish swimming behavior
(a) Time spent at the top, (b) time spent at the bottom, (c) total distance travelled, and (d) freezing time during the NTT are presented for buspirone (10, 30, and 100 mg/L). Black dashed lines = control; light blue = lowest concentration; blue = middle concentration; purple = highest concentration. For anatabine only, red is the highest concentration. *p<0.05; ***p<0.001. Data are expressed as mean ± SEM.
