# Supplementary material for: Effects of nicotinic acetylcholine receptor-activating alkaloids on anxiety-like behavior in zebrafish
Source: J Nat Med. 2021 Jul 15;75(4):926–41. doi: 10.1007/s11418-021-01544-8 (PMC8397634; doi:10.1007/s11418-021-01544-8)
Supplement: Supplementary file 7 — Supplementary file7 (PDF 115 KB) [file 11418_2021_1544_MOESM7_ESM.pdf]

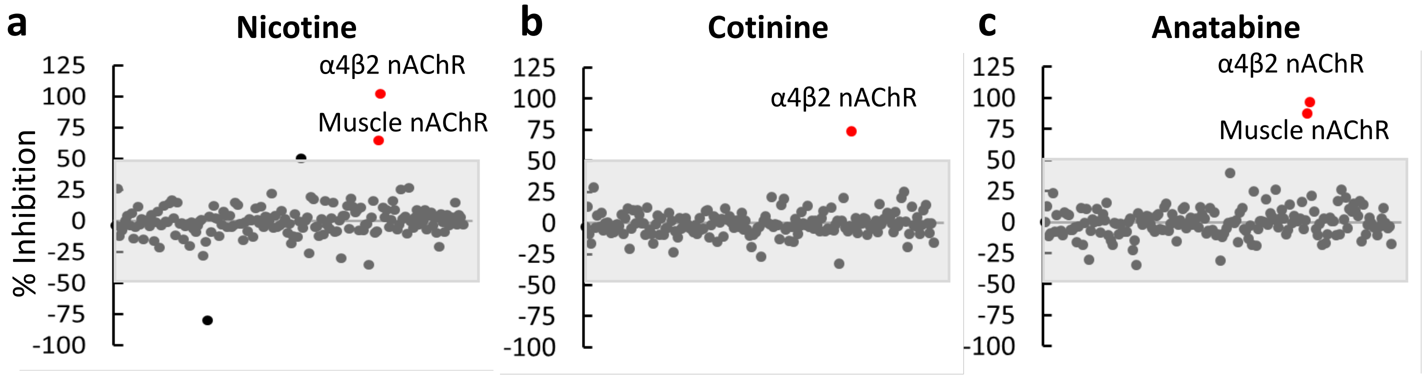

Online Resource 7 Off-target effect assessment

(A) Nicotine, (B) cotinine, and (C) anatabine were tested in 175 in vitro binding or functional assays as summarized in **Online Resource 2.** Each dot represents the outcome of one assay. The grey shaded area covers any assay that showed an effect smaller than 50%. Red dots represent assays that showed greater than 50% change by the respective compounds compared to the vehicle control. N = three repeats.
